# Rapid Androgen-Responsive Proteome Is Involved in Prostate Cancer Progression

**DOI:** 10.3390/biomedicines9121877

**Published:** 2021-12-10

**Authors:** Jong-Kwang Kim, Jae-Hun Jung, Dong-Hoon Shin, Hye-Jin You, Seho Cha, Bo-Seul Song, Jae-Young Joung, Weon-Seo Park, Kwang-Pyo Kim, Jae-Kyung Myung

**Affiliations:** 1 Research Core Center, National Cancer Center, Goyang-si 10408, Korea; jk@ncc.re.kr; 2 Department of Applied Chemistry, Institute of Natural Science, Global Center for Pharmaceutical Ingredient Materials, Kyung Hee University, Yongin-si 17104, Korea; ultramanyo@naver.com; 3 Department of Biomedical Science and Technology, Kyung Hee Medical Science Research Institute, Kyung Hee University, Seoul 02447, Korea; 4 Department of Cancer Biomedical Science, National Cancer Center Graduate School of Cancer Science and Policy, Goyang-si 10408, Korea; dhshin@ncc.re.kr (D.-H.S.); hjyou@ncc.re.kr (H.-J.Y.); sehocha35@gmail.com (S.C.); mawsonyya@naver.com (B.-S.S.); urojy@ncc.re.kr (J.-Y.J.); 5 Research Institute, National Cancer Center, Goyang-si 10408, Korea; 6 Division of Cancer Biology, National Cancer Center, Goyang-si 10408, Korea; 7 Precision Medicine Branch, Research Institute, National Cancer Center, Goyang-si 10408, Korea; 8 Department of Pathology, National Cancer Center, Goyang-si 10408, Korea; thymus@ncc.re.kr

**Keywords:** castration resistant prostate cancer, androgen receptor, rapid signaling, proteomics, mass spectrometry, bioinformatics

## Abstract

Androgen exerts its functions by binding with an androgen receptor (AR). It can activate many signaling pathways that are important to the progression of castration-resistant prostate cancer (CRPC). Here, we characterized the rapid proteomic changes seen at 5, 15, 30, and 60 min after the androgen treatment of VCaP cells via the tandem mass tag (TMT) labeling strategy. A total of 5529 proteins were successfully identified and quantified. Dynamic time profiling of protein expression patterns allowed us to identify five protein clusters involved in various stages of androgen-initiated signal transmission and processing. More details of protein functions and localization patterns, and our elucidation of an AR-interacting protein network, were obtained. Finally, we validated the expression level of AR-regulated proteins known to be significantly regulated in CRPC patients using the mouse xenograft model and patient samples. Our work offers a systematic analysis of the rapid proteomic changes induced by androgen and provides a global view of the molecular mechanisms underlying CRPC progression.

## 1. Introduction

Prostate cancer is the second leading cause of male cancer, and it is one of the most common causes of cancer death (after lung cancer) in the United States [1]. The development of prostate cancer is induced by androgens; thus, androgen-deprivation therapies, such as medical or surgical castration, are used to decrease the physiological androgen level in more than 30% of patients diagnosed with prostate cancer [2,3]. However, most patients treated with androgen-deprivation therapies experience a disease recurrence that is known as castration-resistant prostate cancer (CRPC). One mechanism responsible for the development of CRPC is the acquisition of the metabolic capability to convert steroid precursors to dihydrotestosterone (DHT), which leads to the activation of androgen receptor (AR) signaling [4,5,6,7]. AR, which is a member of the nuclear receptor superfamily, contributes to the development of both normal prostate and prostate cancer. The binding of a ligand to AR can induce the transcription of specific genes via a process that is known to require at least 60 min. However, several studies have shown that various signaling pathways are regulated by androgen stimulation at earlier time points (5 to 60 min), and that this rapid AR signaling contributes to prostate cancer cell proliferation [8,9,10]. The AR-mediated transcription of downstream genes requires a series of processes: DHT binds to AR in the cytoplasm; AR translocates to the nucleus; and the promoters of AR-dependent genes bind to coactivators and/or corepressors of canonical AR signaling. However, increasing evidence shows that the intracellular signaling pathways that contribute to AR-mediated cancer cell proliferation are triggered by a mechanism that is more complex than this canonical signaling [11,12,13]. This mechanism, which is induced at early time points, has been called “rapid” or “non-genomic” signaling and may initiate within seconds after DHT stimulation [14]. Since non-genomic signaling affects various intracellular responses, the analysis of androgen-regulated protein dynamics at early time points might provide insight into yet-unknown mechanisms of prostate cancer development. In addition, the large-scale systematic temporal proteomic analysis of cells under DHT treatment could improve our understanding of the dynamic AR singling network.

Here, we treated VCaP cells with DHT for 5, 15, 30, and 60 min and performed extensive proteomic profiling and quantification by isobaric tandem mass tagging (TMT) analysis. We analyzed changes in the whole proteome at each time point after DHT treatment and clustered proteins that exhibited similar alterations. Such proteins are expected to be involved in cross-talk during DHT-induced AR signaling. Based on the obtained proteomic data, we predicted the molecular characteristics of proteins at each time point, describes a putative protein–protein interaction network, and suggested some candidate targets for the treatment of CRPC. Our results may be used to identify proteins that participate in the early phase of prostate cancer development and provide insight into the protein network that functions under androgen stimulation.

## 2. Materials and Methods

### 2.1. Cell Culture and Protein Sample Preparation

VCaP cells, which represent a human prostate cancer cell line derived from a vertebral metastatic lesion, were obtained from ATCC and grown in Dulbecco’s modified Eagle’s medium (DMEM, HyClone, Logan, UT, USA) supplemented with 10% (*v*/*v*) fetal bovine serum (FBS, HyClone), and 100 units/mL penicillin-streptomycin (Gibco, Carlsbad, CA, USA) at 37 °C in a humidified atmosphere of 5% CO_2_ and 95% air. VCaP cells were serum-starved for 48 h and harvested after treatment with 10 nM dihydrotestosterone (DHT) or vehicle for 5, 15, 30, and 60 min. VCaP cells harvested prior to treatment were used as a control. Proteins were extracted with a RIPA buffer containing 20 mM Tris-HCl, pH 7.5, 150 mM NaCl, 1% NP-40, 0.5% sodium deoxycholate, 1 mM EDTA, 10 mg/mL leupeptin, 10 mg/mL aprotinin, 2 mM _3_VO_4_, 10 mM beta-glycerophosphate, 1 mM PMSF (Roche Applied Science, Mannheim, Germany), PhoSTOP (Roche), and a protease inhibitor cocktail (Roche). The concentration of total proteins was determined with a BCA protein assay kit (Thermo Scientific, Foster City, CA, USA), and aliquots of whole-cell lysates (100 μg) from each sample were used for protein digestion.

### 2.2. Protein Digestion by Filter-Aided Sample Preparation and Tandem Mass Tag Labeling

Briefly, 100 micrograms of protein from each sample type were digested, followed by reduction with SDT buffer (4% SDS in 0.1 M Tris-HCl, pH 7.6, and 0.1 M DTT) at 37 °C. Each protein sample was transferred to a Microcon device (YM-30, Millipore Corporation, Bedford, MA, USA) and mixed with 8 M urea in 0.1 M Tris-HCl, pH 8.5. The protein sample was centrifuged at 14,000× *g* for 60 min with 8 M urea three times to remove the SDS and subjected to alkylation for 25 min with 0.05 M iodoacetamide in 8 M urea at room temperature in the dark. Buffer exchange was performed with 50 mM ammonium bicarbonate and pH 8.0, trypsin (Promega, Madison, WI, USA) was added to the filter at an enzyme-to-protein ratio of 1:50 (*w*/*w*), and the proteins were digested overnight at 37 °C. A second trypsin digestion (1:100 ratio) was carried out at 37 °C for 6 h. All peptides obtained from a given sample were pooled, and the final peptide concentration was determined by the BCA assay. All experiments were performed in triplicate.

Peptides were labeled with the TMT™ Isobaric Mass Tagging reagent according to the manufacturer’s instructions (Thermo Scientific; Foster City, CA, USA). Each sample (100 μg peptides) was mixed with 50 μL of 200 mM triethylammonium bicarbonate (TEAB) and then with TMT reagents that had been resuspended in anhydrous acetonitrile. After 1 h, the reaction was quenched with 5% hydroxylamine. The chemically tagged peptides of each sample (100 μg) were pooled into a single tube and subjected to mid-pH fractionation.

### 2.3. Mid-pH Fractionation and Protein Identification by LC-MS/MS

Mid-pH RPLC (reverse-phase liquid chromatography) was used to separate peptides based on their hydrophobicity. Samples were divided into 15 fractions using an Agilent 1260 series HPLC system (Agilent Technologies, Santa Clara, CA, USA). Briefly, an Accucore™ 150 C_18_ LC Column (150 mm × 2.1 mm, 4 μm) was used for fractionation with a high-pH buffer A, B; 10 mM triethylammonium bicarbonate (TEAB) (pH 7.5) in water as mobile phase (A) and 10 mM TEAB and 90% ACN (pH 7.5) as mobile phase (B). The gradient was as follows: 0–10 min, 5% B; 10–70 min, 35% B; 70–85 min, 70% B; 85–90 min, 5%B; 90–105 min, 5% B. The separated peptides were collected, dried in a speed-vac, and desalted with a C18 spin column (Thermo Scientific).

The desalted and fractionated peptides were resuspended in 0.1% FA and analyzed using a Q-Exactive Orbitrap hybrid mass spectrometer (Thermo Fisher) coupled with an EASY-nLC 1000 system (Thermo Fisher). For proteomic profiling, the gradient (180 min) was as follows: 5–35% solvent B over 150 min; 35–80% solvent B over 1 min; hold at 80% solvent B for 10 min; and equilibrate the column at 1% solvent B for the remainder of the run time. The peptides were eluted through a trap column and ionized through an Easy nano spray analytical column (50 cm × 75 μm ID packed with 2 μm C18 particles; Thermo Scientific) at an electric potential of 2.0 kV. The Q-Exactive Orbitrap mass spectrometer was used to acquire tandem mass spectrometric data. The scan range for full MS (400–2000 Th) was acquired at a resolution of 70,000 (at m/z 400) with an automated gain control (AGC) target value of 1.0 × 10^6^ ions and a maximum ion injection of 120 ms. To investigate low-abundance proteins, we used the DDA (data-dependent acquisition) scan mode, selected the top 10 precursors, and fragmented them into b and y ions in a cycle. The maximal ion injection time for MS/MS was set to 60 ms at a resolution of 17,500. The dynamic exclusion time was set to 30 s. The Thermo Scientific™ Proteome Discoverer™ software version 1.4 was applied to search the obtained MS/MS spectra against the UniProt human database using the SEQUEST HT^®^ search engine. The utilized static modifications included carbamidomethylation (C) and TMT sixplex (N-terminal, K), while the utilized dynamic modifications included methionine oxidation. The resulting peptide hits were filtered for a maximum 1% FDR using the Percolator algorithm [15]. The TMT 10-plex quantification method within the Proteome Discoverer software was used to calculate the reporter ratios with a mass tolerance of ±10 ppm without applying isotopic correction factors. Only peptide spectra containing all reporter ions were designated as “quantifiable spectra”. A protein ratio was expressed as a median value of the ratios for all quantifiable spectra of the peptides pertaining to that protein.

### 2.4. Protein Clustering

The raw ratios representing the expression level of each protein in DHT- versus ethanol-treated cells for each time profile were median-centered, averaged over three technical replications, log2 transformed, and standardized with respect to the zero mean and unit variance across the tested time points. These pre-processed ratio data were fed into a fuzzy c-means (FCM) clustering algorithm implemented in the Mfuzz R-package [16]. In this analysis, noisy patterns and cluster artifacts in time course data can be prevented by optimizing the FCM parameter, *m*. We used a previously described *m* estimation method [17] to calculate that *m* should be set at 2.01. To find the optimal number of clusters, which is unknown *a priori*, we used the elbow method [18,19]. FCM assigned larger membership values (>0.4) to 3454 distinct protein profiles; smaller values represented uncertain (i.e., fuzzy) results, and such proteins were excluded from further analysis.

### 2.5. Protein Subcellular Localization Analysis and Generation of the Protein–Protein Interaction Network

The cellular localization of the identified proteins was annotated according to ingenuity pathway analysis (IPA). IPA annotated the 3454 selected proteins to one of four subcellular localizations: the extracellular space (3.6%), the plasma membrane (10.2%), the cytoplasm (56.6%), and the nucleus (29.5%). The hypergeometric test was used to examine whether each cluster was enriched for proteins of a given subcellular localization. The subcellular distribution was depicted in a strip chart, and the enriched categories were marked (*p*-value < 0.05).

To generate our human PPI network, we used 16,941 direct interactions or physical associations downloaded from BioGrid (https://thebiogrid.org, accessed on 21 March 2019) and generated PPI interaction networks using Cytoscape [20].

### 2.6. Protein-Type Enrichment Analysis

The dynamic patterns were sorted according to their enrichment for the various molecular types, followed by PPI network analysis. For each molecular type, the number of proteins that were sorted to a given profile were tested for enrichment (hypergeometric *p*-value < 0.05). For a given pair of molecular types, each pair of dynamic profile patterns were tested for enrichment in the number of proteins that physically interacted between them (*p*-value < 0.05). The results of these enrichment analyses were used to generate a summarized map of the interactions between the molecular types. Each node represents the molecular type, and a line connects two molecular types if there is more than 1% PPI. A red line signifies a large number of interactions between two profiles (*p*-value < 0.05). The direction of each line was determined according to our findings regarding the signal flow from clusters *A* to *E*.

### 2.7. Signal Cascade Analysis

For each pair of protein clusters, we counted the number of PPIs between their member proteins and used these counts (%) to generate a heatmap. The PPIs found to be overrepresented in cluster pairs *C/D*, *C/E*, and *D/E* were used to construct a signaling network that was displayed on a two-dimensional plane (dynamic profile pattern vs. subcellular compartment). The proteins were distributed to nine separate regions, which were connected according to the human PPI network curated in BioGrid (https://thebiogrid.org, accessed on 21 March 2019). The line directions were drawn in order from *A* to *E,* based on the signal flows revealed in our analysis.

### 2.8. Functional Annotation Enrichment Analysis

For enrichment analysis, proteins in each cluster were searched against seven databases of various types, including gene annotation, gene expression, regulatory element, and pathway databases (Table S2). Each pair of molecular concepts (gene sets) was tested for association using Fisher’s exact test. Each concept was analyzed independently; those that returned significant enrichment (*p*-value < 0.001 and odd ratio > 2) were retained and integrated into a network (concept map). Each node in the map represents a concept, and each line between two nodes depicts a significant association. The node size is proportional to the number of genes in the concept, and the line thickness is proportional to the significance of the association. Concept maps were visualized using Cytoscape [20].

To elucidate the hormone-refractory (or metastatic prostate) marker genes in each cluster, six microarray datasets of prostate cancer were downloaded from the GEO database of genes found to be differentially expressed (up- or down-regulated) in hormone-refractory or metastatic prostate cancer compared to naive prostate (Table S3). All gene expression data were normalized, log-transformed, and median centered per array, and the standard deviation was normalized to one. Each gene was assessed for differential expression using a two-sample *t*-test (significance cutoff level, 0.05). Genes that were up- or down-regulated in at least two independent datasets were considered to be marker genes.

TCGA prostate mRNA-seq data (provisional) obtained from 52 paired prostate adenocarcinoma and matched normal samples were downloaded from cBioPortal [21,22]. The data were obtained as normalized estimate values (level 3) from Illumina HiSeq/GA2, and a heatmap was generated using the z-scores of the normalized expression. A two-sample *t*-test was used to identify genes that were differentially expressed (up-/down-regulated) in adenocarcinoma (*p*-value < 0.01).

### 2.9. Luciferase Assay and BrdU Incorporation Assays

Transcriptional activity of the AR was determined in VCaP cells transfected with PSA promoter-luc and FASN-specific small interfering RNA (siRNA). Cells treated with androgen (R1881) for 72 h were lysed and assayed for luciferase activities using the Dual-Luciferase Reporter Assay System (Promega).

The BrdU incorporation assay was performed using the Cell Proliferation ELISA, BrdU (colorimetric) kit (Roche Applied Science) to measure cell proliferation in the presence and absence of FASN siRNA. VCaP cells treated with R1881 for 72 h were labeled and fixed. Absorbance was measured at 450 nm, and ELISA BrdU was performed according to the manufacturer’s protocol. All data represent the mean (±standard deviation, SD) of three independent experiments (^##^ *p* < 0.01, ^###^ *p* < 0.001, ** *p* < 0.01.).

### 2.10. Immunohistochemistry Analysis

Immunohistochemistry was performed using tumor tissues obtained from male mice xenografted with 10 million VCaP cells. This study was reviewed and approved by the Institutional Animal Care and Use Committee (IACUC) of the National Cancer Center Research Institute. Tumor volume was measured with calipers every 3–4 days. At 53 days after injection, intact mice of the control group were sacrificed, while mice of the CRPC group were castrated and monitored until sacrifice. Sectioned specimens were incubated with anti-FASN (at 1:100, Thermo Fisher) overnight at 4 °C. The specimens were blocked, washed, and then incubated with a biotinylated secondary antibody (1:200; Vector Laboratories, Burlingame, CA, USA) for 1 h. The immune complexes were visualized using a VECTASTAIN ABC kit (Vector Laboratories, USA), and the sections were counterstained with hematoxylin, dried, and mounted with DAKO aqueous mounting solution (Agilent Technologies, Santa Clara, CA, USA).

## 3. Results

### 3.1. FASP-Coupled TMT Labeling Provides Quantitative Proteomic Information on the Response That Is Rapidly Induced by Androgen in VCaP Cells

**Figure 1:**
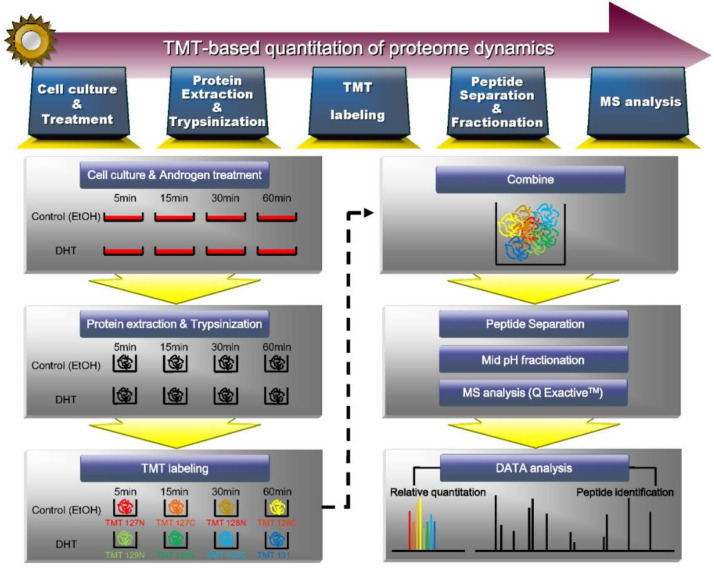
Experimental scheme for the identification and quantification of proteome rapidly induced by androgen. Proteins were extracted from VCaP cells treated with DHT or vehicle for 5, 15, 30, and 60 min and digested with trypsin. A total of peptides were labeled with TMT labeling reagents and identified and quantified by mass spectrometry.

To gain insight into the proteome dynamics rapidly induced by dihydrotestosterone (DHT, a representative physiologic androgen hormone) in prostate cancer, we investigated the protein changes that occur within 60 min of DHT treatment. We selected VCaP cells, which are widely used for the study of CRPC progression, and treated these cells without any reagent (untreated control, time point 0) or with DHT or ethanol (treatment control) for 5, 15, 30, and 60 min. Cell proteins were extracted, digested using the Filter-aided Sample Preparation (FASP) method, and subjected to tandem mass tag (TMT) labeling for proteomic quantitation. The experimental scheme is shown in Figure 1. The TMT isobaric labeling reagents utilize the 6-mDa mass difference between the 13C and 15N isotopes, as measured by high-resolution LC-MS/MS.

A total of 5529 proteins were successfully identified and quantified in triplicate at each of four time points (5, 15, 30, and 60 min) with more than two unique peptides (*q* value < 0.01) and 4532 proteins commonly identified in all three experiments were accepted for further analysis to obtain functional information (Figure S1A). To confirm the reproducibility of the experiment, we performed principal component analysis (PCA) and determined the protein expression profile in triplicate at each of the four time points (5, 15, 30, and 60 min). We found that the triplicate results obtained at each time point were clustered together. The batch effect between replicates was the smallest for the 60-min time point (Figure S1B).

### 3.2. Androgen Treatment Triggers Dynamic Changes of the Proteome in VCaP Cells

**Figure 2:**
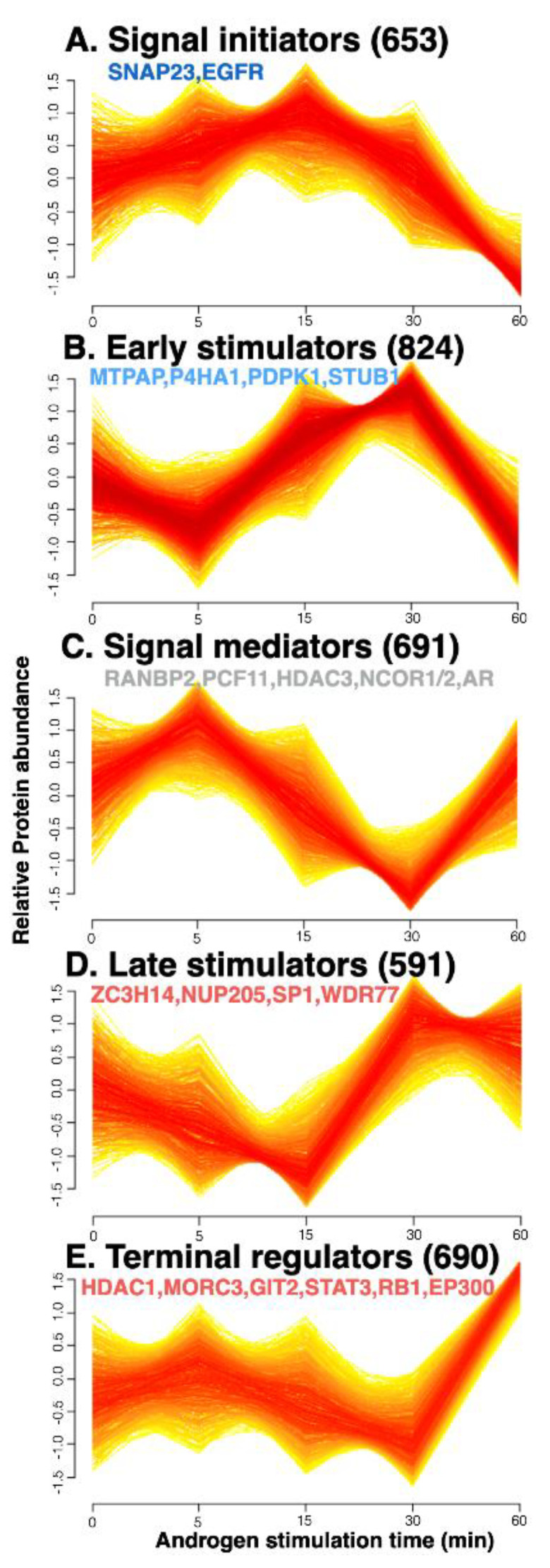
Proteins were clustered by their dynamic responses to androgen in VCaP prostate cancer cells. Based on their androgen-response time profiles, proteins were assigned membership values using a fuzzy c−means clustering algorithm. Each trace is colored according to its membership value. All profiles presented here are of high membership value (>0.4). The generated protein clusters represent proteins that showed up-regulation in the early time points (**A**,**B**), and in the late time points (**D**,**E**), and both in the early and late time points (**C**).

Numerous proteins and multiple pathways can be involved in the downstream effects of androgen stimulation, regardless of whether the action is genomic or non-genomic. To systematically analyze the dynamic protein changes that occur during the non-genomic rapid androgen response, proteins that exhibited similar expression patterns over time were clustered by a fuzzy c-means (FCM) clustering algorithm that filtered out noisy profiles. We predicted the optimal number of clusters, which is unknown *a priori*, using the elbow method (Figure S2), and obtained five clusters of proteins (Figure 2). These included two clusters of the up-regulated proteins in the early time points (A and B), which were termed “signal initiators: and “early stimulators”, respectively. In addition, two clusters termed “late stimulators” and “terminal regulators” represented up-regulated proteins in the late time points (D and E, respectively), and one of signal mediator proteins which are up-regulated both in the early and late time points (C).

### 3.3. Time-Related Androgen-Response Profiles of Proteins Correlate with Their Subcellular Localizations

**Figure 3:**
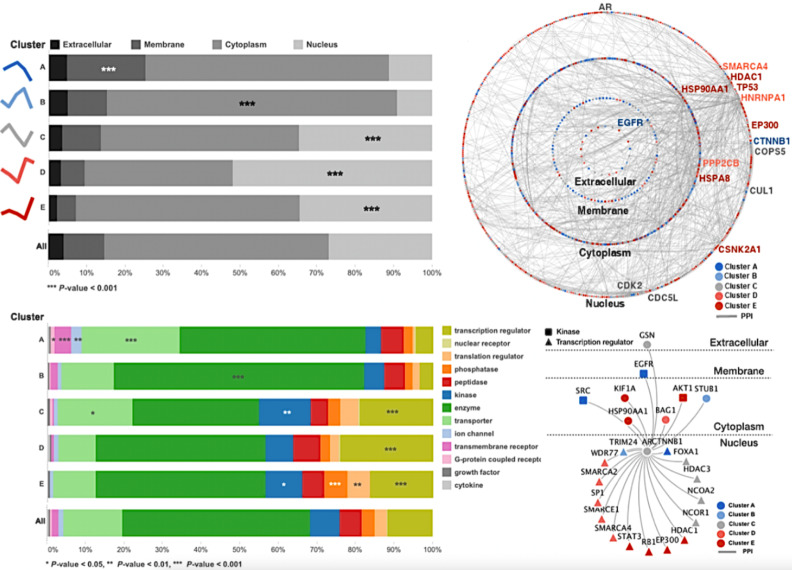
Androgen-response profiles correlate with the subcellular localization of the proteins. (**A**) Relative proportions of protein localization for each cluster show that cluster *A* is enhanced for plasma membrane proteins (hypergeometric *p*-value < 0.001), whereas its up-regulated counterpart cluster, *E*, is enhanced for nuclear proteins. The clusters are ordered according to their proportions of plasma membrane proteins. (**B**) The spectra of protein types for each cluster show that the type-enrichment patterns are associated with the androgen-response profile. Clusters of proteins that change in different directions (e.g., **A**,**E**) show mutually exclusive enrichment patterns. (**C**) All clustered proteins are laid out in circles according to their cellular compartments and are colored by their clusters. The down-regulated proteins (**A**,**B**) are enriched for plasma membrane proteins (blue), whereas the up-regulated proteins (**D**,**E**) are enriched for nuclear proteins (red). The oscillating proteins do not show any bias for a given compartment. The hub protein, EGFR, directly interacts with AR and with various downstream proteins in different cellular compartments. (**D**) Neighboring proteins of AR in the high-confidence protein–protein interaction network are presented (1st and 2nd only).

We next investigated whether proteins with similar androgen-response profiles might colocalize in subcellular compartments. Information of subcellular compartments of proteins in each cluster was obtained by ingenuity pathway analysis (IPA) and represented in Figure 3. Interestingly, higher-than-expected proportions of proteins of a given cluster were found to colocalize in certain compartments (*p*-value < 0.001) (Figure 3A). The membrane and cytoplasmic proteins were enriched in clusters with up-regulated proteins in the early time points (*Cluster A* and *B*), whereas nuclear proteins were enriched in signal mediator clusters (*Cluster C*) and two of up-regulated proteins in the late time points (*Cluster D* and *E*).

Different clusters exhibited different protein-type enrichment patterns. Our IPA annotated the 3454 selected proteins to 13 molecular types: transcription regulators, ligand-dependent nuclear receptors, translation regulators, phosphatases, peptidases, kinases, enzymes, transporters, ion channels, transmembrane receptors, G-protein coupled receptors, growth factors, and cytokines. A hypergeometric cutoff *p*-value of 0.05 was used to identify enriched protein types (Figure 3B). For example, cluster *A* was enriched for plasma membrane proteins (20.5%) as shown in Figure 3A, many of which were transporters (25.4%) and transmembrane receptors (4.3%) with the significance of *p*-values of less than 0.001. These proteins were therefore termed “signal initiators”. In contrast, cluster *E* was enhanced for nuclear proteins (34.5%), including many transcriptional (16.4%) and translational regulators (5.9%), which were termed “terminal regulators”.

Inspired by the apparent correlation between the localizations and types of the enriched proteins of each cluster, we next examined how AR-interacting proteins might relate to the obtained androgen-response profiles. To achieve this goal, we used our identified proteome data in comparison to the public database to generate a high-confidence protein–protein interaction (PPI) network with an intra- and inter-molecular interaction of proteins in each cluster observed in different subcellular compartments. A global view of the proteome interactions of each cluster and the network of AR interactions for each cluster was further examined (Figure 3C,D). Most proteins identified in our study have interacts with EGFR, which are known to interact with the AR. EGFR physically interacted with 97 other proteins and thus functions as the main hub protein of this androgen-signaling network. Notably, EGFR showed 1.5 (=45/29) times more interaction with up-regulated proteins in the late time points (late stimulators or terminal regulators) than in the early time points (signal initiators or early stimulators), as also seen in the enrichment of the cytoplasm in Figure 3C. TP53, which is one of the most popular proteins involved in castration-resistant prostate cancer progression, interacted with 87 proteins and was the second most important hub protein. All proteins physically interacting with EGFR and TP53 found in VCaP cells elucidated by integrative analysis of PPI network and our proteome data were listed in Table S1, and the most top 15 high-degree hub proteins and AR were represented in a Figure 3C.

In addition, our results indicated that in our system, most changes triggered by DHT would occur through AR, as AR was shown to directly interact with various proteins, including three “signal initiators” (CTNNB1, EGFR, and SRC), two “early stimulators” (STUB1 and TRIM24), five “signal mediators” (GSN, FOXA1, HDAC3, NCOA1/2), six “late stimulators” (BAG1, SMARCA2/4, SMARCE1, SP1, and WDR77), and seven “terminal regulators” (AKT1, EP300, HDAC1, RB1, STAT3, HSP90AA1, and KIF1A), (Figure 3D).

### 3.4. An AR Signaling Network Generated from the Functional Categories of AR-Regulated Proteins Reveals the Molecular Interactions of AR-Responsive Proteins

**Figure 4:**
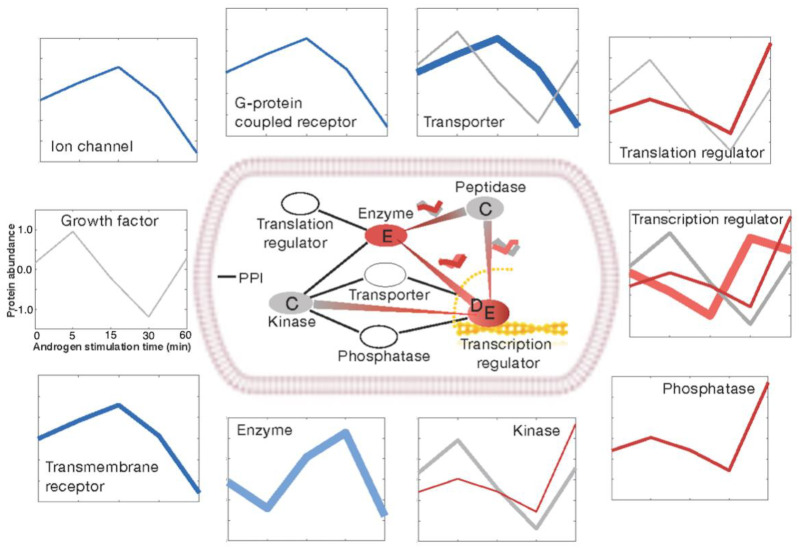
Functional categories of the androgen-responsive proteome. The regulated proteins fall into 10 major functional categories, as shown in the outer squares. The consensus kinetic curves of its members, as shown in clustering analysis, were drawn in proportion to the number of proteins in each cluster. The clusters that have a significantly large number of proteins were only presented (*p*-value < 0.05). In the inner square, the protein–protein interactions between functional categories are displayed. A line is drawn between any two categories if more than 5% of their member proteins interact. The red line signifies a large number of interactions between two clusters (*p*-value < 0.05).

We sorted the androgen-regulated proteins according to their molecular types (Figure 4) and found that they could be grouped into two major classes. One major class comprised ion channels, transmembrane receptors, G-protein coupled receptors, enzymes, and transporters (shown as blue lines in the outer squares of Figure 4). The main kinetic clusters in these protein categories were the signal initiators and early stimulators, which were consistently down-regulated in the late time points under androgen treatment. The second major class comprised translation/transcription regulators, phosphatases, and kinases (shown as red lines in the outer squares of Figure 4). These proteins were enhanced for late stimulators and terminal regulators, which showed increased expression over time under androgen stimulation. Our results reveal that the androgen signal can spread to transcriptional regulators within the short time frame of 60 min.

**Figure 5:**
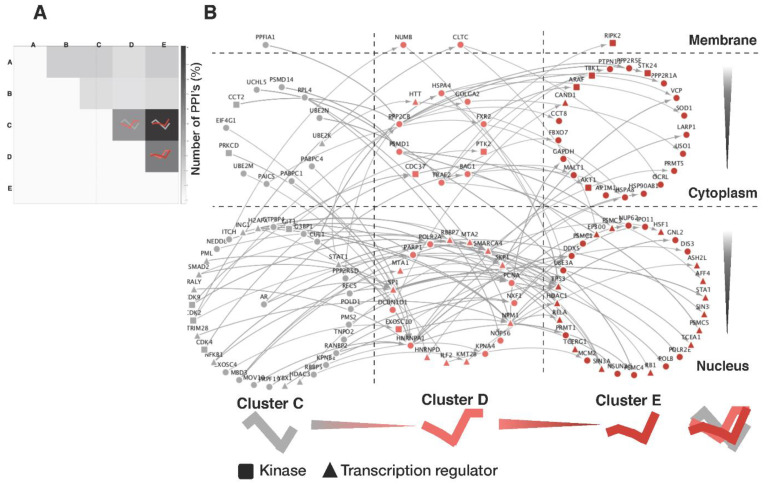
Signaling networks in different cellular compartments. (**A**) A heatmap represented the numbers of interacting protein pairs from each pair of clusters. Our systematic analysis revealed that the cluster pairs *C/E*, *D/E*, and *C/D* are over-represented. The proteins of these clusters are likely to represent major signaling partners in the response of VCaP prostate cancer cells to androgen, as may be exemplified by the well-known interaction between AR (cluster *C*) and SP1 (cluster *D*). (**B**) Systematic enumeration and visualization of the signaling proteins that connect cluster *C* to cluster *E* via cluster *D* in the context of their cellular compartments.

The apparent association between dynamic protein patterns and their subcellular localizations motivated us to systematically analyze signaling cascades in the PPI network. To examine the extent to which the proteins of a specific dynamic pattern interacted with proteins of different dynamics, we counted the interactions among all possible pairs of interacting proteins belonging to any two different clusters. Consistent with the results of our AR signaling network analysis generated from the functional categories of androgen-regulated proteins, as shown in the inner square of Figure 4, the interactions between members of clusters *C* and *E* outnumbered those of the other combinations (Figure 5A). Moreover, three cluster pairs (*C/E*, *D/E*, and *C/D*) accounted for 64.4% of all inter-signaling links, implying that these proteins are the major mode through which androgen-induced signaling is propagated in VCaP cells. For these cluster pairs, we constructed a network of signaling proteins based on their subcellular localization and known PPIs and all 139 signaling cascades, including AR signaling shown in Figure 5B. As could be expected based on the results of our time-related androgen-response profiling analysis in the correlation with their subcellular localizations, many kinase proteins were located in the cytoplasm, and many transcriptional regulators were located in the nucleus. We listed the 47 signaling cascades (from *C* to *E* via *D*) that extend from upstream transcriptional regulators to downstream transcriptional regulators that interact with the final target proteins (Table 1).

### 3.5. The Most Representative Proteome in Each Cluster Correlates with Clinical Outcome Information

**Figure 6:**
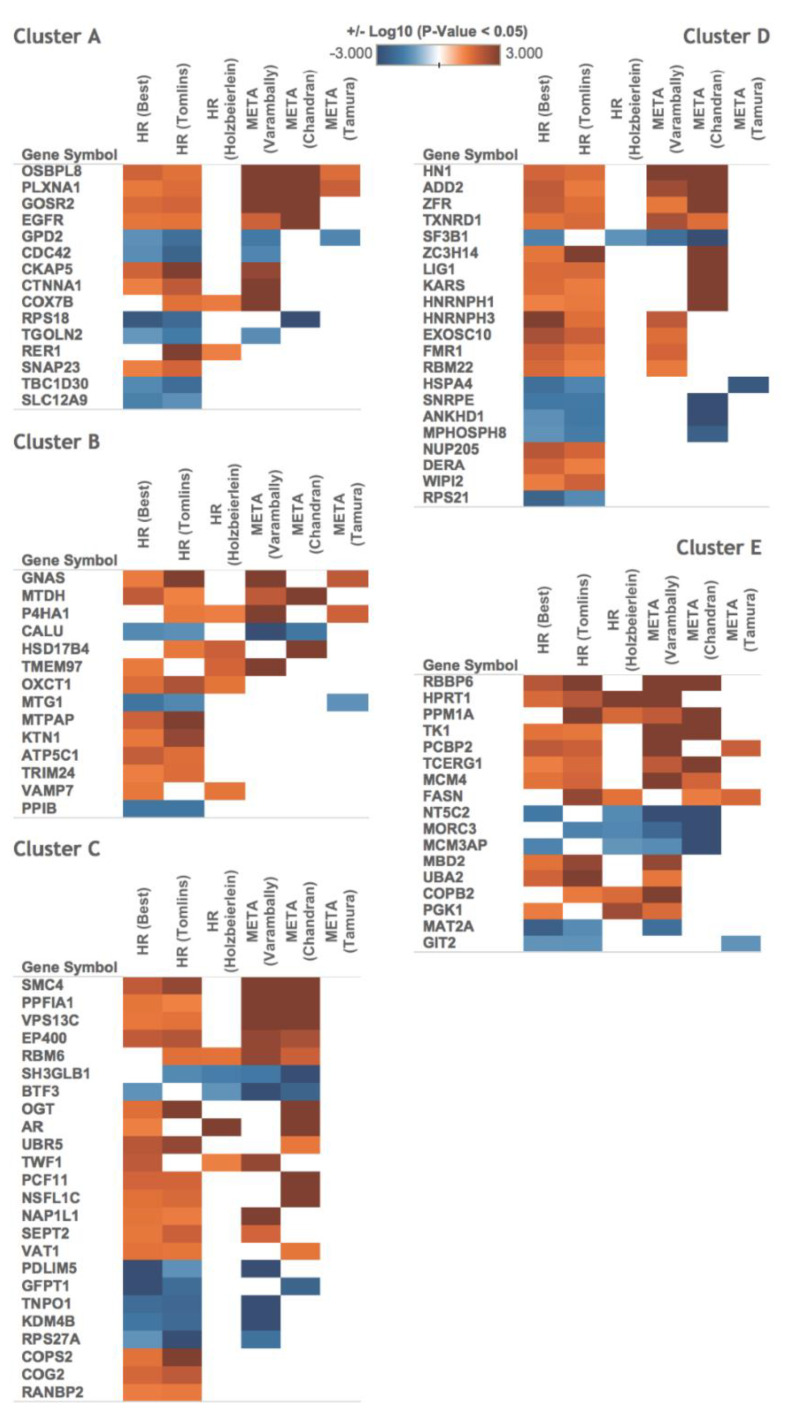
The proteins of the identified androgen-response profiles are associated with hormone-refractory prostate cancer and metastasis. The proteins of each cluster (cluster A, B, C, D, and E) were functionally characterized by searching an open database (Oncomine) for known marker genes. The analysis revealed that our clusters include many signature genes previously reported to be significantly up-/down-regulated (*p*-value < 0.05) in hormone-refractory (HR) or metastatic (META) prostate cancer. Genes that exhibited differential expression in at least two datasets are presented.

We herein used VCaP cells, which are widely applied in studying the progression of CRPC. To get a clinical insight into our findings, we identified relevant datasets and tested whether proteins of each cluster are differentially expressed in clinical samples. We searched the Oncomine database of microarray data (https://www.oncomine.org, accessed on 25 March 2020) for representative genes known to show differential expression at the mRNA level in hormone-refractory or metastatic prostate cancer and then compared their encoded proteins to our clustered proteins. We found that 91 genes previously shown to be altered under hormone-refractory or metastatic prostate cancer encoded proteins that exhibited changes following androgen stimulation of VCaP cells (Figure 6). We further examined the 91 putative marker genes in an independent prostate cancer dataset from the cancer genome atlas (TCGA) and found that 24 of the 91 marker genes were up-/down-regulated in prostate adenocarcinoma vs. matched normal samples (*p*-value < 0.01), as shown in (Figure S3). Among these genes, the encoded products of 21 (87.5%) were involved in binding activities (GO enrichment test, *p*-value < 0.001), especially cadherin binding (SH3GLB1, CKAP5, and FASN), manganese ion binding (MTPAP and PPM1A), methylated histone binding (MPHOSPH8 and TRIM24), protein homodimerization activity (FASN and HSD17B4), and identical protein binding (CDC42 and TK1).

Interestingly, fatty acid synthase (FASN) was up-regulated in our late time point datasets which are designed as an androgen-responsive terminal regulator (cluster *E*). We have further investigated and obtained expression and functional information of FASN in prostate cancer progression (Figure S4). FASN is the key enzyme for the control of fatty acid synthesis that contributes significantly to prostate cancer progression, and FASN was observed to be relatively high in castrated mouse xenograft tissues compared to intact ones (Figure S4A). The level of FASN protein expression in prostate cancer patient tissues was increased according to the increase of the grade and higher TNM stages (Figure S4B), as shown in the prostate adenocarcinoma primary solid tumor from Illumina mRNAseq level_3 (v2) data set (Figure S4C). Androgen increased expression of FASN at both mRNA and protein levels, as shown in Figure S4D. In addition, the knock-out of FASN gene expression by siRNA process reduced androgen-induced prostate cancer cell proliferation (left) and transcriptional activity of the AR (right) in VCaP prostate cancer cells (Figure S4E).

### 3.6. A Molecular Concept Map of the Androgen-Responsive Proteome Provides Functional Information

We extensively characterized our androgen-responsive proteome by using a molecular concept map to explore the relationships among the biologically related gene sets of seven databases and 340 high-throughput prostate cancer datasets. We compared each protein cluster with all concepts in the collection and used disproportionate overlaps to identify a network of linked concepts (Figure S5).

Several lines of functional information obtained from this analysis support the evidence that characterized our cluster profiles. As an example, cluster *A* (653 signal initiators) was associated with the most highly enriched literature concept, “up-regulated genes (time-dependent) in prostate cancer cells in response to androgen” (*p*-value = 1.02 × 10^−15^, odds ratio = 7.4) and with the second most highly enriched concept, “up-regulated genes in prostate cancer cells in response to synthetic androgen R1881” (*p*-value = 1.2 × 10^−10^, odds ratio = 5.1). The most highly enriched biological process among our signal initiators was “protein transport” (*p*-value = 5.25 × 10^−19^, odds ratio = 7.1), and this was consistent with the results of our independent analysis of the subcellular localization of the androgen-responsive proteins (Figure 3B). Oxidative phosphorylation was the most highly enriched KEGG pathway in cluster *A* (*p*-value = 6.41 × 10^−29^, odds ratio = 12.3) (Figure S5A). Thus, our results suggest that androgen is likely to trigger a major signaling initiation process through oxidative phosphorylation and certain transport proteins, including CDC42 (cell division cycle 42), LMAN1/2 (lectin, mannose-binding 1/2), MTX1 (metaxin1), NUP88 (nucleoporin), PREB (prolactin regulatory element-binding), RAB (RAS oncogene family member), RHOA/B (ras homolog gene family member A/B), SCAMP1/2/4 (secretory carrier membrane protein1/2/4), and RASEF (RAS and EF-hand domain-containing). As another example, cluster *E* (690 terminal regulators), which has a profile inverse to that of cluster *A*, contains a number of genes that were underexpressed in LNCaP cells subjected to R1881 treatment and AR transfection (*p*-value = 7.73 × 10^−11^, odds ratio = 2.6) (Figure S5E). The biological processes enriched in cluster *E* included protein biosynthesis (*p*-value = 2.18 × 10^−12^, odds ratio = 4.2) and protein folding (*p*-value = 9.26 × 10^−10^, odds ratio = 4.2).

### 3.7. Development of PCaDB (Prostate Cancer DataBase) to Provide Comprehensive Information Regarding Dynamic Proteome Events in Prostate Cancer

Our quantitative data on the dynamic proteomic changes triggered by androgen treatment of VCaP cells is a useful resource for studying the progression of CRPC. To enable these data to be efficiently used by the research community, we constructed a web-accessible database called PCaDB (http://52.78.16.249 (accessed on 21 March 2019)) (Figure S6). PCaDB lists the dynamic time profiles of the 4532 identified proteins and provides an interactive visual map for the major signaling cascades, extending from signal mediators to terminal regulators via late stimulators (Figure 5B). The data are cross-referenced to information in UniProt, HGNC, and PDB.

## 4. Discussion

In the present study, we analyzed the proteomic changes that occurred rapidly (within 60 min) in androgen-treated prostate cancer cells. We observed changes in many proteins that are associated with distinct biological functions and cellular compartments and clustered such proteins by their patterns of abundance at each time point.

Canonical AR-mediated signaling (also known as the genomic signaling pathway) occurs via the binding of androgen with AR and the subsequent nuclear translocation of AR. This alters AR-mediated gene expression over the course of several hours. In contrast, the non-genomic signaling triggered by androgen occurs much more rapidly. For that reason, most researchers have examined translational modifications (e.g., changes in phosphorylation) when seeking to elucidate immediate-early responses under androgen stimulation. However, Costello et al. showed that both prolactin and testosterone regulate the transcriptional level of m-aconitase within 15 min in prostate cells [23]. Similarly, the ubiquitin proteasome system-mediated protein degradation induced by inhibition of mTOR was shown to occur within 30 min [24]. It has also been suggested that protein abundances at early time points following external stimulation might be effected via direct and/or indirect mechanisms. In view of these reports, we herein examined the overall proteomic profiles for the early response of prostate cancer cells to DHT and sought to predict the putative functional roles of the identified proteins in prostate cancer progression.

As expected, we did not observe dramatic changes of the proteome in cellular expression levels throughout the time points, but information of significantly different cellular compartments and biological functions in accordance with protein abundances at each time points was revealed by m-fuzz-based clustering results (Figure 2). We next used computational analysis to determine an AR-interacting protein network (Figure 3D). Our results showed that AR could interact with highly abundant proteins at immediate-early time points (within 30 min), including STUB1, SRC, and GSN, as well as with abundant proteins at later time points (30–60 min), such as EP300, SP1, and STAT3. Notably, the latter proteins can contribute to transcriptional activity. Our results suggest that information regarding significant differences in protein abundance at each early time point might provide insight into the immediate cellular responses triggered by androgen stimulation in prostate cancer cells.

Notably, the signaling network and protein interaction results presented in Figure 5 show that there was a relatively high proportion of protein–protein interactions among clusters *C*–*E* compared to clusters *A* and *B*. This suggests that abundant proteins altered at early time points play relatively intrinsic roles (e.g., protein modification) in the androgen-mediated signaling cascade rather than participating in the cascade through complex formation. Consistent with these results, our molecular concept map (Figure S5) indicates that proteins of cluster *B* were associated with metabolic functions, while those of cluster *E* participated in chromatin remodeling and splicing. Although we do not yet clearly understand how the expression levels of these proteins are regulated at early time points following androgen stimulation, these changes could logically involve cellular processes such as the alteration of protein stability by protein modification, the facilitation of translational processing, and the activation or inactivation of rapid autophagy. The regulatory role of androgen as well as AR signaling in the autophagic process has been already known as one of the key mechanisms involved in the transition of prostate cancer cells from an androgen-dependent to an androgen-independent cell type [25,26], and targeting autophagy was observed to overcome Enzalutamide resistance in CRPC [27].

Of the proteins identified by our early abundance profiling, the encoding genes of 91 were listed in the Oncomine database as being differentially expressed in prostate carcinoma and hormone-refractory prostate cancer (Figure 6), and the encoding genes of 24 were listed in the TCGA database as being differentially expressed in prostate adenocarcinoma (Figure S3). Since FASN expression is correlated with a high Gleason score for prostate cancer, these results suggest that the proteins selected in the present study, which we identified using a computational analysis based on protein abundance at early time points, might play functional roles in prostate cancer progression.

As shown in Figure S4, we confirmed the differential expression levels and further provided functional information of FASN in prostate cancer progression via in-vitro, in-vivo and clinical studies. The well-known prostate cancer marker gene, FASN, had a good distinction between tumor and normal patient samples in TCGA mRNA-seq as well as microarray data and our present proteome datasets. FASN was dramatically increased in CRPC mouse tissues (Figure S4A) and prostate cancer patient tissues (Figure S4B), as shown in the expression signature of prostate cancer patients (Figure S4C) [28]. Androgen increased expression of FASN mRNA and protein levels (Figure S4D) and prostate cancer cell proliferation, and AR transcriptional activity was shown to be related with the FASN expression as shown in Figure S4E. The biological relevance of these changes will be further investigated in a future study. FASN and AR were previously detected in 87% of metastases in human mCRPC [29]. FASN-mediated prostate cancer progression was suggested as a clue about the failure of the current androgen deprivation therapy, and the inhibition of FASN-mediated signaling was represented as a new therapeutic approach to suppress prostate cancer progression during the castration-resistant stage of prostate cancer [30]. Future studies are needed to examine whether FASN protein could be targeted for therapeutic efforts against prostate cancer, but the collected data indicate that FASN mediates the effect of cell proliferation, and these results suggest that increased expression of FASN significantly contributes to the increased invasive potential of prostate cancer cells and targeting FASN would be potential benefits for the clinical use.

## 5. Conclusions

We herein gained insight into the proteomic alterations seen at early time points following the exposure of prostate cancer cells to androgen and used our findings to generate a cascade network that connects the immediate-early and early-abundant proteins in prostate cancer cells. Our findings could help improve the current understanding of the intracellular responses induced by androgen and provide novel perspectives on the role of rapid androgen signaling in CRPC progression with high potential therapeutic benefits.

## Figures and Tables

**Table 1:** List of proteins and molecular types involved in androgen responsive signaling cascades of VCaP cells that extend from upstream signal mediators (cluster C) to late stimulators (cluster D) interacting with downstream transcriptional regulators (cluster E).

Protein_C	Protein_D	Protein_E	Type_C	Type_D	Type_E
UBE2N	TRAF2	AFF4	Enzyme	Enzyme	Tc Reg ^1^
RBBP5	KMT2B	ASH2L	Enzyme	Tc Reg	Tc Reg
PMS2	PCNA	EP300	Enzyme	Enzyme	Tc Reg
POLD1			Enzyme		
RFC5			Enzyme		
KPNB1	KPNA4	HDAC1	Transporter	Transporter	Tc Reg
PMS2	PCNA	HDAC1	Enzyme	Enzyme	Tc Reg
POLD1			Enzyme		
RFC5			Enzyme		
STAT1	SMARCA4	HDAC1	Tc Reg	Tc Reg	Tc Reg
STAT1	SMARCA4	HSF1	Tc Reg	Tc Reg	Tc Reg
STAT1	SMARCA4	RB1	Tc Reg	Tc Reg	Tc Reg
GTPBP4	NPM1	RELA	Enzyme	Tc Reg	Tc Reg
H2AFX			Tc Reg		
H2AFX	PARP1	RELA	Tc Reg	Enzyme	Tc Reg
NFKB1			Tc Reg		
MBD3	MTA2	SIN3A	Enzyme	Tc Reg	Tc Reg
ING1	RBBP7	SIN3A	Tc Reg	Tc Reg	Tc Reg
MBD3			Enzyme		
STAT1	SMARCA4	SIN3A	Tc Reg	Tc Reg	Tc Reg
ING1	RBBP7	SIN3B	Tc Reg	Tc Reg	Tc Reg
MBD3			Enzyme		
AR	SP1	STAT3	LD NR ^2^	Tc Reg	Tc Reg
NFKB1			Tc Reg		
PML			Tc Reg		
PRKCD			Kinase		
SMAD2			Tc Reg		
CDK9	POLR2A	TCEA1	Kinase	Enzyme	Tc Reg
ITCH			Enzyme		
CDK9	POLR2A	TCERG1	kinase	Enzyme	Tc Reg
ITCH			Enzyme		
G3BP1	HNRNPA1	TP53	Enzyme	Enzyme	Tc Reg
MBD3	MTA1	TP53	Enzyme	Tc Reg	Tc Reg
MBD3	MTA2		Enzyme	Tc Reg	
GTPBP4	NPM1	TP53	Enzyme	Tc Reg	Tc Reg
H2AFX			Tc Reg		
H2AFX	PARP1	TP53	Tc Reg	Enzyme	Tc Reg
NFKB1			Tc Reg		
AR	SMARCA4	TP53	LD NR	Tc Reg	Tc Reg
H2AFX			Tc Reg		
HDAC3			Tc Reg		
SMAD2			Tc Reg		
AR	SP1	TP53	LD NR	Tc Reg	Tc Reg
NFKB1			Tc Reg		
PML			Tc Reg		
PRKCD			Kinase		
SMAD2			Tc Reg		

^1^ Tc Reg, Transcription regulator; ^2^ LD NR, ligand-dependent nuclear receptor.

## Supplementary Materials

The following are available online at https://www.mdpi.com/article/10.3390/biomedicines9121877/s1, Figure S1: The number of total proteome identified from the experiments in triplicate and principal component analysis (PCA) plot provide a reproducibility of the proteome analysis, Figure S2: Determine the optimal number of clusters in proteome data set by cluster variance curve, Figure S3: Analysis of mRNA expression levels of differentially expressed genes was determined in TCGA prostate cancer samples, Figure S4: Expression of FASN was associated with prostate cancer progression in tumor tissue from both CRPC mouse xenografts and patient samples and functional information of FASN was validated in prostate cancer cell, Figure S5: Molecular concept map provides an overview of biological relevance with each cluster associated with each cluster, Figure S6: PCaDB (Prostate Cancer Database) is a useful resource to provide dynamic proteomic changes triggered by androgen in VCaP cells, Table S1: List of proteins interacting with EGFR or TP53 in VCaP cells, Table S2: Types of molecular concept class used for enrichment analysis, Table S3: Details of protein annotation datasets of hormone refractory or metastatic prostate cancer used in this study for functional enrichment analysis of clusters.
