# Supplementary material for: Rapid Androgen-Responsive Proteome Is Involved in Prostate Cancer Progression
Source: Biomedicines. 2021 Dec 10;9(12):1877. doi: 10.3390/biomedicines9121877 (PMC8698566; doi:10.3390/biomedicines9121877)
Supplement: Supplementary file 1 [file biomedicines-09-01877-s001.zip › Table S1.pdf]

Table S1. List of proteins interacting with EGFR or TP53 in VCaP cells

Hub Protein	HGNCSymbol	UniProt_SwissProtAccession	Cluster	Location
**EGFR**	ATP1A1	P05023	A	Plasma Membrane
	CAMLG	P49069	A	Cytoplasm
	CTNNB1	P35222	A	Nucleus
	CTNND1	O60716	A	Nucleus
	FRK	P42685	A	Nucleus
	RPN1	P04843	A	Cytoplasm
	SEC61B	P60468	A	Cytoplasm
	SGPL1	O95470	A	Cytoplasm
	SRC	P12931	A	Cytoplasm
	TIMM50	Q3ZCQ8	A	Cytoplasm
	ACOT9	Q9Y305	B	Cytoplasm
	ATP2A2	P16615	B	Cytoplasm
	ATP5A1	P25705	B	Cytoplasm
	ATP5C1	P36542	B	Cytoplasm
	CANX	P27824	B	Cytoplasm
	CDH1	P12830	B	Plasma Membrane
	DNAJA3	Q96EY1	B	Cytoplasm
	HSP90B1	P14625	B	Cytoplasm
	HSPA5	P11021	B	Cytoplasm
	IGF1R	P08069	B	Plasma Membrane
	LYN	P07948	B	Cytoplasm
	PAK1	Q13153	B	Cytoplasm
	PON2	Q15165	B	Plasma Membrane
	PTPN1	P18031	B	Cytoplasm
	SEC61A1	P61619	B	Cytoplasm
	SLC3A2	P08195	B	Plasma Membrane
	SPTLC1	O15269	B	Cytoplasm
	STUB1	Q9UNE7	B	Cytoplasm
	VAPA	Q9P0L0	B	Plasma Membrane
	AHNAK	Q09666	C	Nucleus
	AR	P10275	C	Nucleus

	ARHGEF7	Q14155	C	Cytoplasm
	BAIAP2	Q9UQB8	C	Plasma Membrane
	CRKL	P46109	C	Cytoplasm
	CTTN	Q14247	C	Plasma Membrane
	DNAAF5	Q86Y56	C	Cytoplasm
	DNAJA1	P31689	C	Nucleus
	DNAJA2	O60884	C	Nucleus
	EIF2B2	P49770	C	Cytoplasm
	EIF2B5	Q13144	C	Cytoplasm
	H1FX	Q92522	C	Nucleus
	HGS	O14964	C	Cytoplasm
	KPNB1	Q14974	C	Nucleus
	PDCD6IP	Q8WUM4	C	Cytoplasm
	PLCG1	P19174	C	Cytoplasm
	PTPN6	P29350	C	Cytoplasm
	SMC2	O95347	C	Nucleus
	SNX2	O60749	C	Cytoplasm
	STAM2	O75886	C	Cytoplasm
	STAT1	P42224	C	Nucleus
	TUBB6	Q9BUF5	C	Cytoplasm
	WASL	O00401	C	Cytoplasm
	ACTN4	O43707	D	Cytoplasm
	AP1B1	Q10567	D	Cytoplasm
	AP1G1	O43747	D	Cytoplasm
	AP1S1	P61966	D	Cytoplasm
	AP2A1	O95782	D	Cytoplasm
	AP2A2	O94973	D	Cytoplasm
	AP2B1	P63010	D	Plasma Membrane
	AP2S1	P53680	D	Cytoplasm
	BCLAF1	Q9NYF8	D	Nucleus
	CALM1	P62158	D	Cytoplasm
	CDC37	Q16543	D	Cytoplasm
	CRK	P46108	D	Cytoplasm
	EIF2B3	Q9NR50	D	Cytoplasm

	HNRNPH1	P31943	D	Nucleus
	HSPA4	P34932	D	Cytoplasm
	ITSN2	Q9NZM3	D	Cytoplasm
	JUP	P14923	D	Plasma Membrane
	LRP1	Q07954	D	Plasma Membrane
	NUP93	Q8N1F7	D	Nucleus
	PTK2	Q05397	D	Cytoplasm
	TRAF2	Q12933	D	Cytoplasm
	AKT1	P31749	E	Cytoplasm
	AP1M1	Q9BXS5	E	Cytoplasm
	ARRB1	P49407	E	Cytoplasm
	CDK1	P06493	E	Nucleus
	CSK	P41240	E	Cytoplasm
	GAPDH	P04406	E	Cytoplasm
	HSP90AA1	P07900	E	Cytoplasm
	HSP90AB1	P08238	E	Cytoplasm
	HSPA8	P11142	E	Cytoplasm
	IPO11	Q9UI26	E	Nucleus
	MMS19	Q96T76	E	Nucleus
	PFKL	P17858	E	Cytoplasm
	PFKP	Q01813	E	Cytoplasm
	PRDX1	Q06830	E	Cytoplasm
	PRKACA	P17612	E	Cytoplasm
	PTPN11	Q06124	E	Cytoplasm
	RBM10	P98175	E	Nucleus
	SNX9	Q9Y5X1	E	Cytoplasm
	STAT3	P40763	E	Nucleus
	TAB1	Q15750	E	Cytoplasm
	USP8	P40818	E	Cytoplasm
	XPO7	Q9UIA9	E	Nucleus
	YWHAB	P31946	E	Cytoplasm
	YWHAZ	P63104	E	Cytoplasm
**TP53**	BAK1	Q16611	A	Cytoplasm
	BCL2L1	Q07817	A	Cytoplasm

	PTTG1IP	P53801	A	Nucleus
	RPL26	P61254	A	Cytoplasm
	RPS26	P62854	A	Cytoplasm
	EHMT1	Q9H9B1	B	Nucleus
	HSPA5	P11021	B	Cytoplasm
	HSPA9	P38646	B	Cytoplasm
	LRPPRC	P42704	B	Cytoplasm
	STUB1	Q9UNE7	B	Cytoplasm
	SYVN1	Q86TM6	B	Cytoplasm
	TRIM24	O15164	B	Nucleus
	CCT2	P78371	C	Cytoplasm
	CCT4	P50991	C	Cytoplasm
	CDK2	P24941	C	Nucleus
	COPS5	Q92905	C	Nucleus
	DNAJA1	P31689	C	Nucleus
	GNL3	Q9BVP2	C	Nucleus
	HNRNPM	P52272	C	Nucleus
	ING1	Q9UK53	C	Nucleus
	ITCH	Q96J02	C	Nucleus
	MAGED2	Q9UNF1	C	Plasma Membrane
	MED1	Q15648	C	Nucleus
	MSH2	P43246	C	Nucleus
	PABPC1	P11940	C	Cytoplasm
	PML	P29590	C	Nucleus
	PPP1R13L	Q8WUF5	C	Nucleus
	PSME3	P61289	C	Cytoplasm
	RBBP5	Q15291	C	Nucleus
	RPL27	P61353	C	Cytoplasm
	RPL8	P62917	C	Cytoplasm
	RPS10	P46783	C	Cytoplasm
	RPS7	P62081	C	Cytoplasm
	SMAD2	Q15796	C	Nucleus
	TP53BP1	Q12888	C	Nucleus

	TRIM28	Q13263	C	Nucleus
	TUBA1C	Q9BQE3	C	Cytoplasm
	TUBB	P07437	C	Cytoplasm
	TUBB	P07437	C	Cytoplasm
	UBE2N	P61088	C	Cytoplasm
	UBR5	O95071	C	Nucleus
	YBX1	P67809	C	Nucleus
	CHEK2	O96017	D	Nucleus
	CREB1	P16220	D	Nucleus
	HNRNPA1	P09651	D	Nucleus
	HSPA4	P34932	D	Cytoplasm
	MTA1	Q13330	D	Nucleus
	MTA2	O94776	D	Nucleus
	NPM1	P06748	D	Nucleus
	NUMB	P49757	D	Plasma Membrane
	PARP1	P09874	D	Nucleus
	PDCD5	O14737	D	Nucleus
	RPLP0	P05388	D	Cytoplasm
	SMARCA4	P51532	D	Nucleus
	SP1	P08047	D	Nucleus
	TOP1	P11387	D	Nucleus
	VRK1	Q99986	D	Nucleus
	AGO1	Q9UL18	E	Cytoplasm
	ARIH2	O95376	E	Nucleus
	BAG2	O95816	E	Cytoplasm
	CCT3	P49368	E	Cytoplasm
	CCT5	P48643	E	Cytoplasm
	CCT6A	P40227	E	Cytoplasm
	CCT7	Q99832	E	Cytoplasm
	CCT8	P50990	E	Cytoplasm
	CDK1	P06493	E	Nucleus
	CSNK2A1	P68400	E	Nucleus
	DDX5	P17844	E	Nucleus

	EP300	Q09472	E	Nucleus
	HDAC1	Q13547	E	Nucleus
	HMGB1	P09429	E	Nucleus
	HSP90AA1	P07900	E	Cytoplasm
	HSPA8	P11142	E	Cytoplasm
	MAPK1	P28482	E	Cytoplasm
	MAPK14	Q16539	E	Cytoplasm
	MAPK3	P27361	E	Cytoplasm
	NFYB	P25208	E	Nucleus
	OTUB1	Q96FW1	E	Cytoplasm
	PSMC5	P62195	E	Nucleus
	RAD23A	P54725	E	Nucleus
	SETD7	Q8WTS6	E	Nucleus
	SIN3A	Q96ST3	E	Nucleus
	TCP1	P17987	E	Cytoplasm
	TK1	P04183	E	Cytoplasm
	UBE3A	Q05086	E	Nucleus
	UCHL1	P09936	E	Cytoplasm
	VCP	P55072	E	Cytoplasm
