# Supplementary material for: Rapid Androgen-Responsive Proteome Is Involved in Prostate Cancer Progression
Source: Biomedicines. 2021 Dec 10;9(12):1877. doi: 10.3390/biomedicines9121877 (PMC8698566; doi:10.3390/biomedicines9121877)
Supplement: Supplementary file 1 [file biomedicines-09-01877-s001.zip › Table S2.pdf]

Table S2. List of proteins and molecular types involved in androgen responsive signaling cascades*.

Protein_C	Protein_D	Protein_E	Type_C	Type_D	Type_E
UBE2N	TRAF2	AFF4	enzyme	enzyme	transcription regulator
RBBP5	KMT2B	ASH2L	enzyme	transcription regulator	transcription regulator
PMS2	PCNA	EP300	enzyme	enzyme	transcription regulator
POLD1			enzyme		
RFC5			enzyme		
KPNB1	KPNA4	HDAC1	transporter	transporter	transcription regulator
PMS2	PCNA	HDAC1	enzyme	enzyme	transcription regulator
POLD1			enzyme		
RFC5			enzyme		
STAT1	SMARCA4	HDAC1	transcription regulator	transcription regulator	transcription regulator
STAT1	SMARCA4	HSF1	transcription regulator	transcription regulator	transcription regulator
STAT1	SMARCA4	RB1	transcription regulator	transcription regulator	transcription regulator
GTPBP4	NPM1	RELA	enzyme	transcription regulator	transcription regulator
H2AFX			transcription regulator		
H2AFX	PARP1	RELA	transcription regulator	enzyme	transcription regulator
NFKB1			transcription regulator		
MBD3	MTA2	SIN3A	enzyme	transcription regulator	transcription regulator
ING1	RBBP7	SIN3A	transcription regulator	transcription regulator	transcription regulator
MBD3			enzyme		
STAT1	SMARCA4	SIN3A	transcription regulator	transcription regulator	transcription regulator

ING1	RBBP7	SIN3B	transcription regulator	transcription regulator	transcription regulator
MBD3			enzyme		
AR	SP1	STAT3	ligand-dependent nuclear receptor	transcription regulator	transcription regulator
NFKB1			transcription regulator		
PML			transcription regulator		
PRKCD			kinase		
SMAD2			transcription regulator		
CDK9	POLR2A	TCEA1	kinase	enzyme	transcription regulator
ITCH			enzyme		
CDK9	POLR2A	TCERG1	kinase	enzyme	transcription regulator
ITCH			enzyme		
G3BP1	HNRNPA1	TP53	enzyme	enzyme	transcription regulator
MBD3	MTA1	TP53	enzyme	transcription regulator	transcription regulator
MBD3	MTA2		enzyme	transcription regulator	
GTPBP4	NPM1	TP53	enzyme	transcription regulator	transcription regulator
H2AFX			transcription regulator		
H2AFX	PARP1	TP53	transcription regulator	enzyme	transcription regulator
NFKB1			transcription regulator		
AR	SMARCA4	TP53	ligand-dependent nuclear receptor	transcription regulator	transcription regulator
H2AFX			transcription regulator		
HDAC3			transcription regulator		

SMAD2			transcription regulator		
AR	SP1	TP53	ligand-dependent nuclear receptor	transcription regulator	transcription regulator
NFKB1			transcription regulator		
PML			transcription regulator		
PRKCD			kinase		
SMAD2			transcription regulator		

* List of proteins and molecular types involved in androgen responsive signaling cascades of VCaP cells was shown extending from upstream signal mediators (cluster C) to late stimulators (cluster D) interacting with downstream transcriptional regulators (cluster E)
