# Supplementary material for: Rapid Androgen-Responsive Proteome Is Involved in Prostate Cancer Progression
Source: Biomedicines. 2021 Dec 10;9(12):1877. doi: 10.3390/biomedicines9121877 (PMC8698566; doi:10.3390/biomedicines9121877)
Supplement: Supplementary file 1 [file biomedicines-09-01877-s001.zip › Table S3_Rev.pdf]

biomedicines

MDPI

Table S3. Details of protein annotation datasets of hormone refractory or metastatic prostate cancer used in this study for functional enrichment analysis of clusters

Prostate cancer data	Study description	Accession
Best *et al*. [31]	Twenty (20) primary prostate adenocarcinoma samples, including 10 hormone refractory and 10 hormone naive, were analyzed.	GSE2443
Chandran *et al*. [32]	Thirty-one (31) prostate carcinoma tissue specimens (10 primary and 21 hormone-refractory metastatic) were analyzed.	GSE6752
Holzbeierlein *et al*. [33]	Fifty (50) prostate carcinoma and 4 normal prostate gland samples were analyzed.	Journal website
Tamura *et al*. [34]	Twenty-five (25) hormone-refractory prostate carcinoma tissue specimens (including 12 metastatic and 13 primary specimens) and 10 hormone-sensitive prostate carcinoma tissue specimens were analyzed.	GSE6811
Tomlins *et al*. [35]	101 laser dissected cell populations from a progression of prostate cancer cell types (benign, PIN, low-grade PCA, high-grade PCA, metastatic PCA) and normal adjacent tissue were analyzed.	GSE6099
Varambally *et al*. [36]	Six (6) hormone-refractory metastatic prostate carcinoma samples, 7 primary prostate carcinoma samples, and 6 normal prostate gland samples were analyzed.	GSE3325

31. Best, C.J.; Gillespie, J.W.; Yi, Y.; Chandramouli, G.V.; Perlmutter, M.A.; Gathright, Y.; Erickson, H.S.; Georgevich, L.; Tangrea, M.A.; Duray, P.H.; et al. Molecular Alterations in Primary Prostate Cancer After Androgen Ablation Therapy. *Clin. Cancer Res*. **2005**, *11*, 6823–6834.
32. Chandran, U.R.; Ma, C.; Dhir, R.; Bisceglia, M.; Lyons-Weiler, M.; Liang, W.; Michalopoulos, G.; Becich, M.; Monzon, F.A. Gene Expression Profiles of Prostate Cancer Reveal Involvement of Multiple Molecular Pathways in the Metastatic Process. *BMC Cancer* **2007**, *7*, 1–21.
33. Holzbeierlein, J.; Lal, P.; LaTulippe, E.; Smith, A.; Satagopan, J.; Zhang, L.; Ryan, C.; Smith, S.; Scher, H.; Scardino, P.; et al. Gene Expression Analysis of Human Prostate Carcinoma during Hormonal Therapy Identifies Androgen-Responsive Genes and Mechanisms of Therapy Resistance. *Am. J. Pathol*. **2004**, *164*, 217–227.
34. Tamura, K.; Furihata, M.; Tsunoda, T.; Ashida, S.; Takata, R.; Obara, W.; Yoshioka, H.; Daigo, Y.; Nasu, Y.; Kumon, H.; et al. Molecular Features of Hormone-Refractory Prostate Cancer Cells by Genome-Wide Gene Expression Profiles. *Cancer Res*. **2007**, *67*, 5117–5125.
35. Tomlins, S.A.; Mehra, R.; Rhodes, D.R.; Cao, X.; Wang, L.; Dhanasekaran, S.M.; Kalyana-Sundaram, S.; Wei, J.T.; Rubin, M.A.; Pienta, K.J.; et al. Integrative Molecular Concept Modeling of Prostate Cancer Progression. *Nat. Genet*. **2007**, *39*, 41–51.
36. Varambally, S.; Yu, J.; Laxman, B.; Rhodes, D.R.; Mehra, R.; Tomlins, S.A.; Shah, R.B.; Chandran, U.; Monzon, F.A.; Becich, M.J.; et al. Integrative Genomic and Proteomic Analysis of Prostate Cancer Reveals Signatures of Metastatic Progression. *Cancer Cell*. **2005**, *8*, 393–406.
