# Supplementary figures and images for: Rapid Androgen-Responsive Proteome Is Involved in Prostate Cancer Progression
Source: Biomedicines. 2021 Dec 10;9(12):1877. doi: 10.3390/biomedicines9121877 (PMC8698566; doi:10.3390/biomedicines9121877)

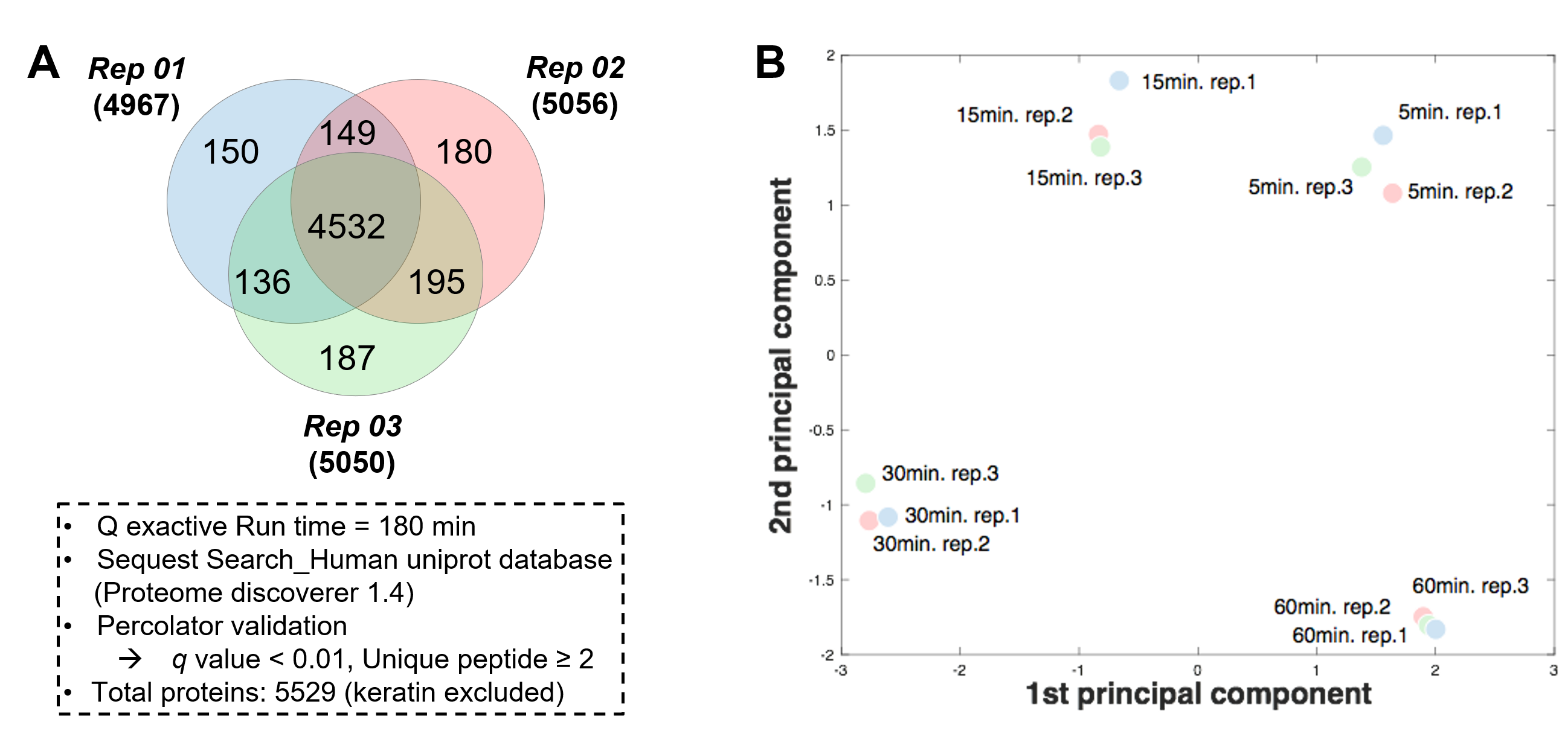

Supplement: Supplementary file 1 [file biomedicines-09-01877-s001.zip › Figure S1.tif]

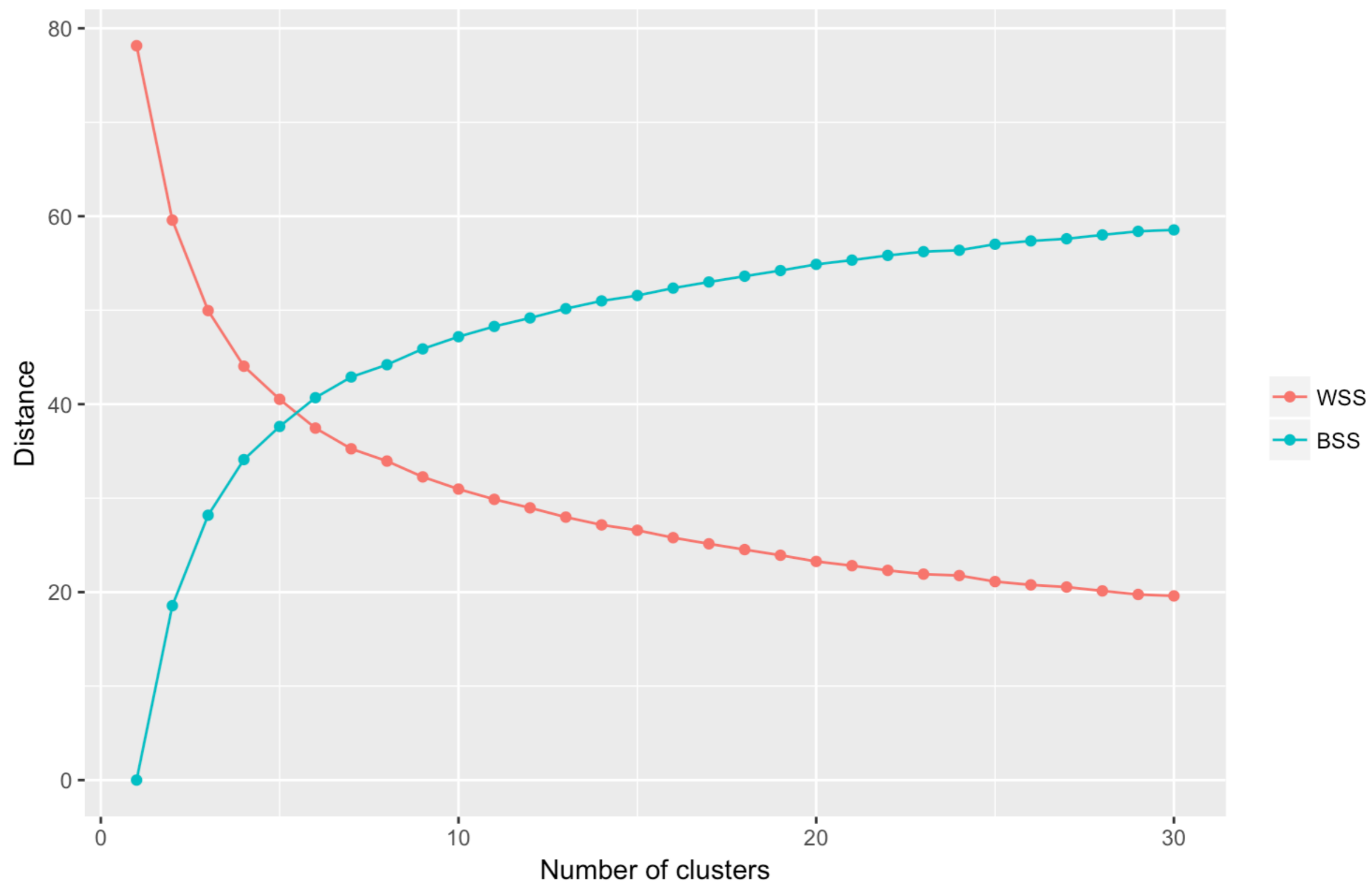

Supplement: Supplementary file 1 [file biomedicines-09-01877-s001.zip › Figure S2.pdf]

cluster

Tumor

Normal

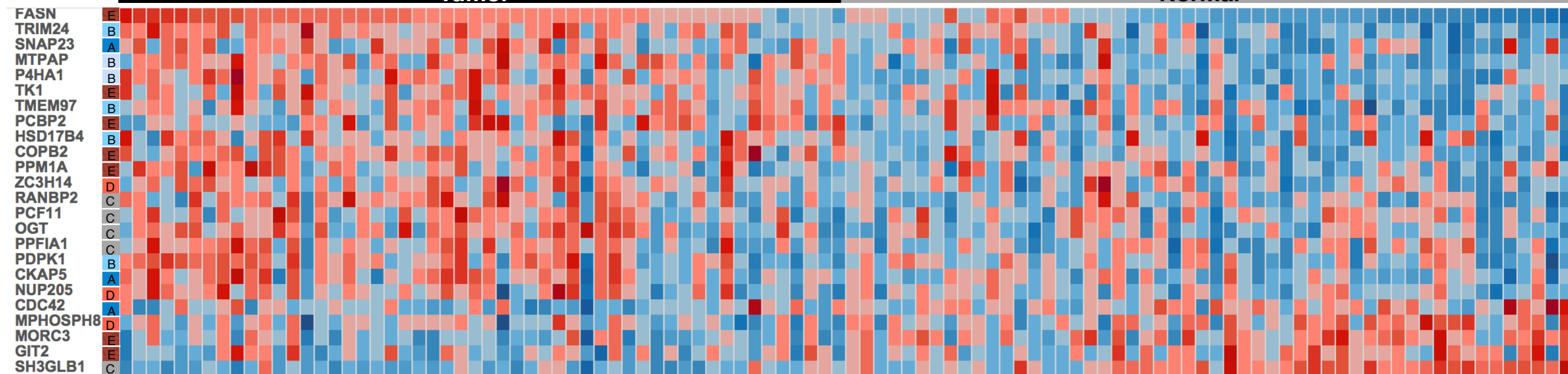

Expression (z-score)

-4.0 4.0

Supplement: Supplementary file 1 [file biomedicines-09-01877-s001.zip › Figure S3.pdf]

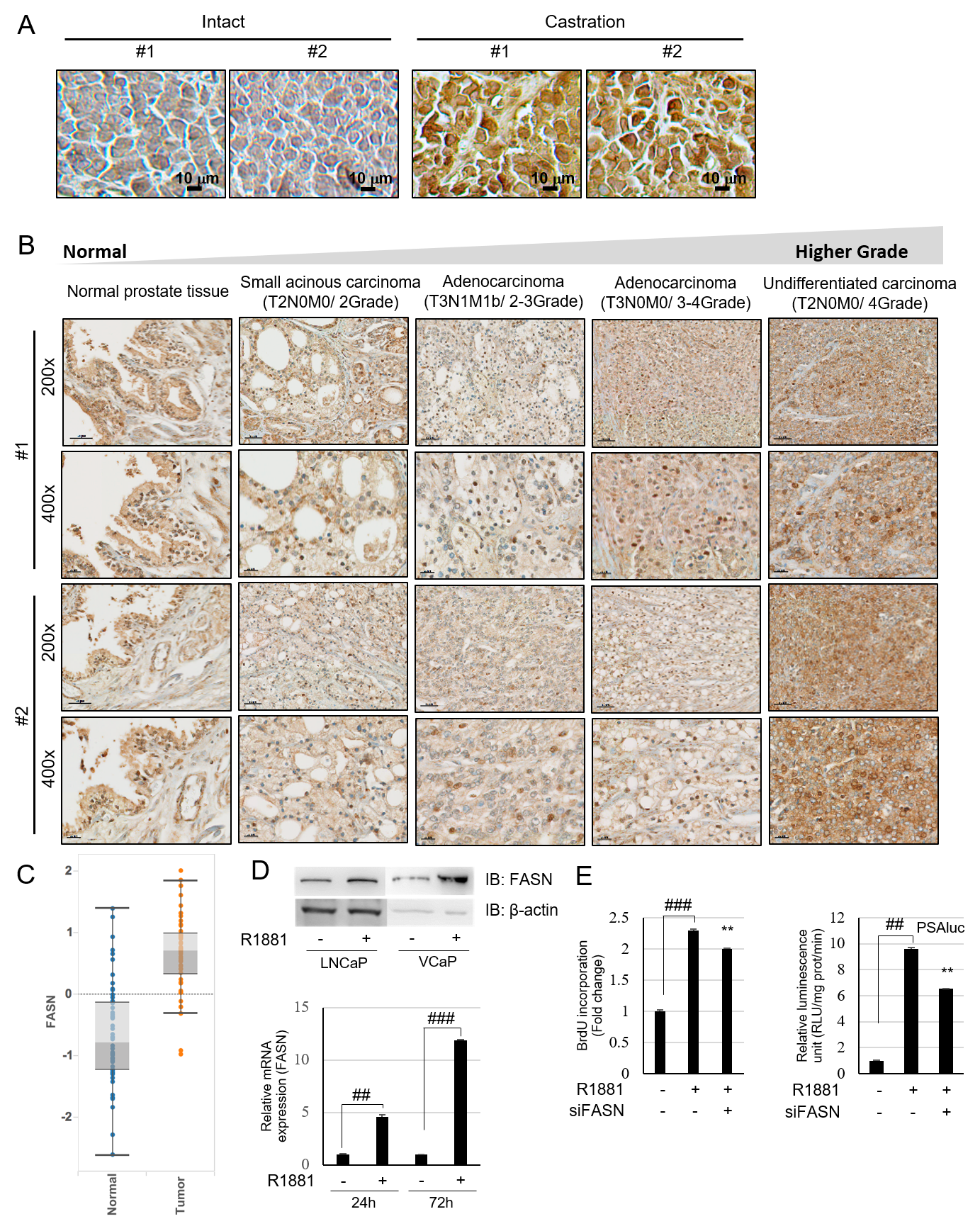

Supplement: Supplementary file 1 [file biomedicines-09-01877-s001.zip › Figure S4.tif]

A

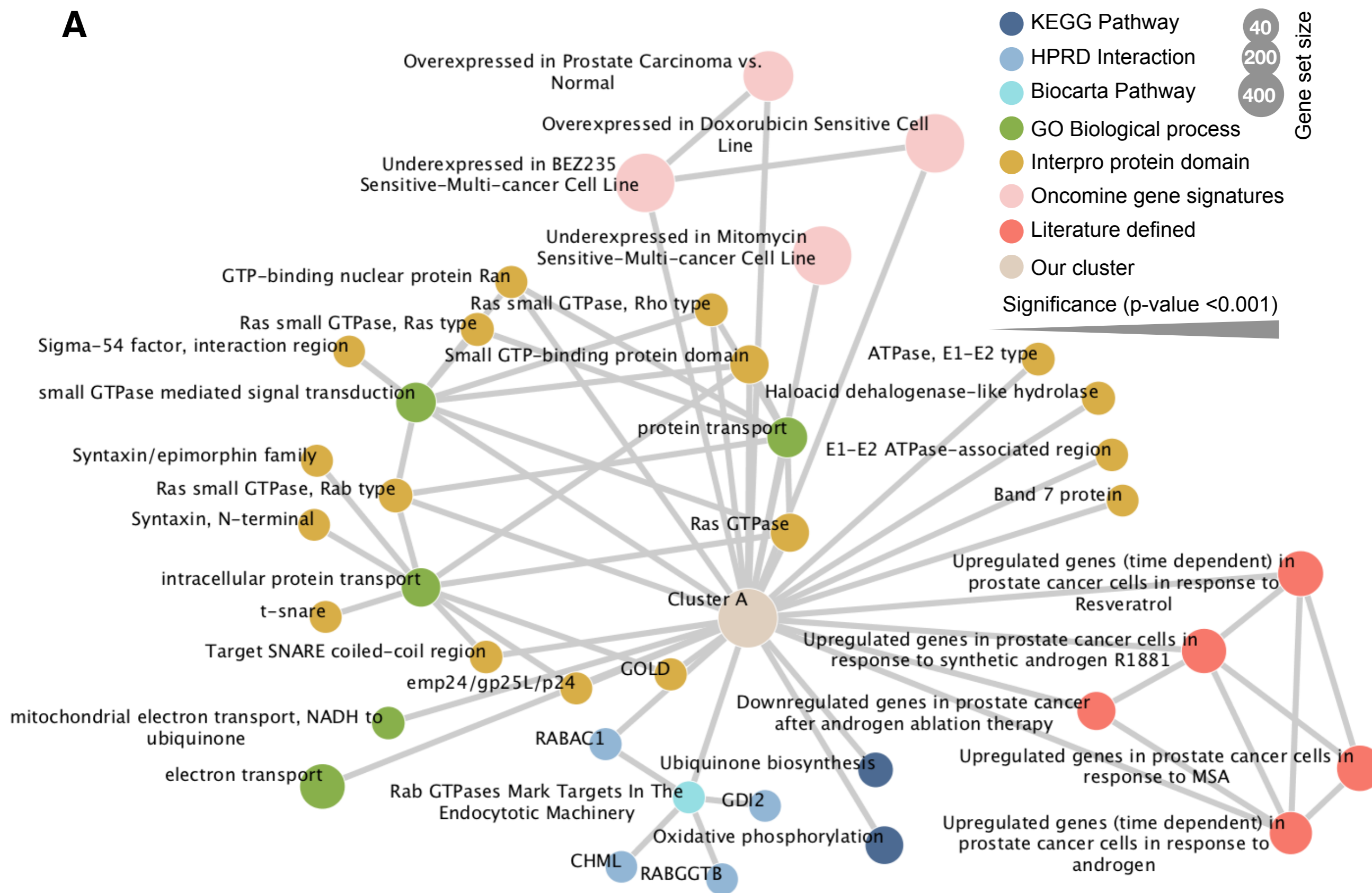

**B**

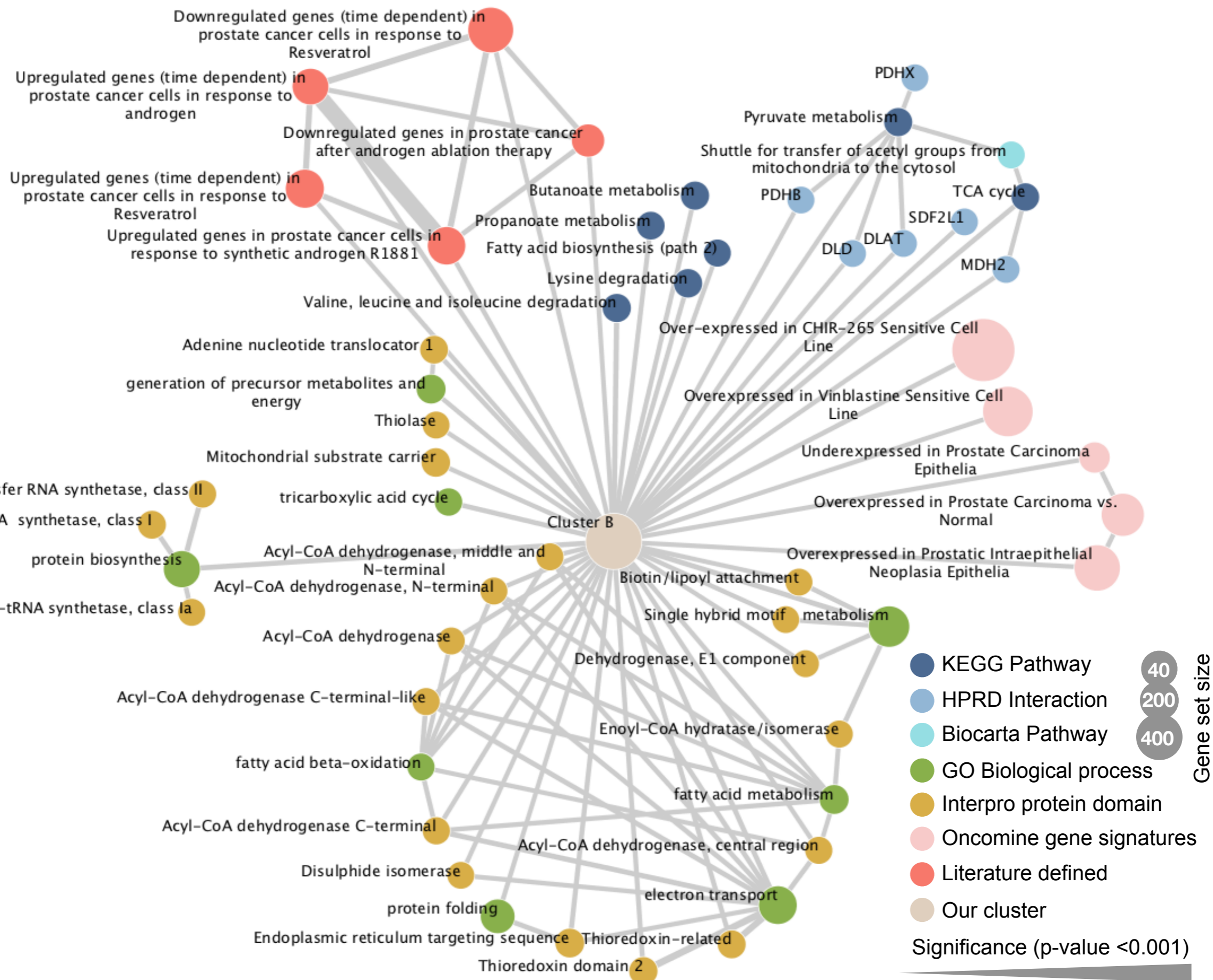

C

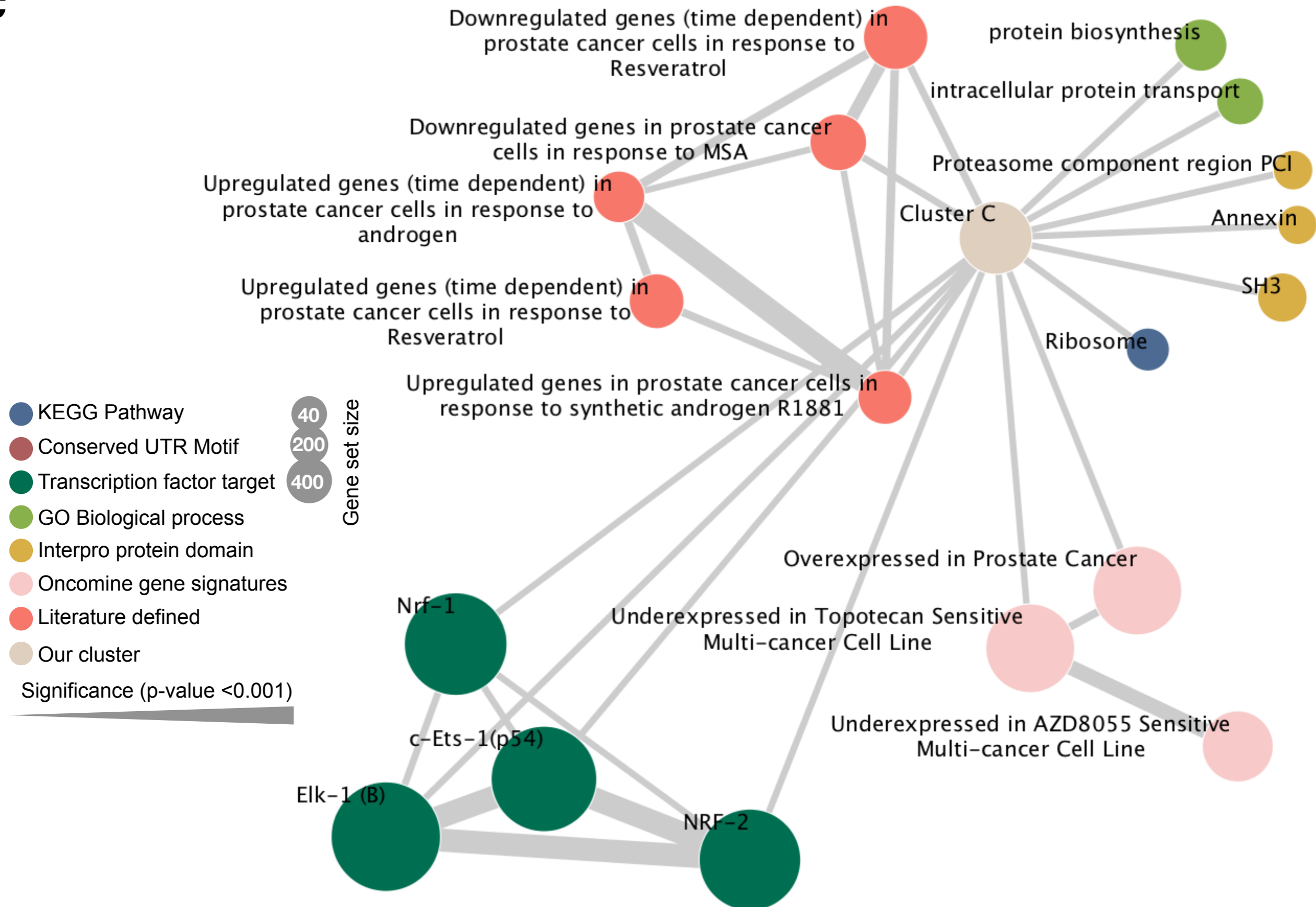

D

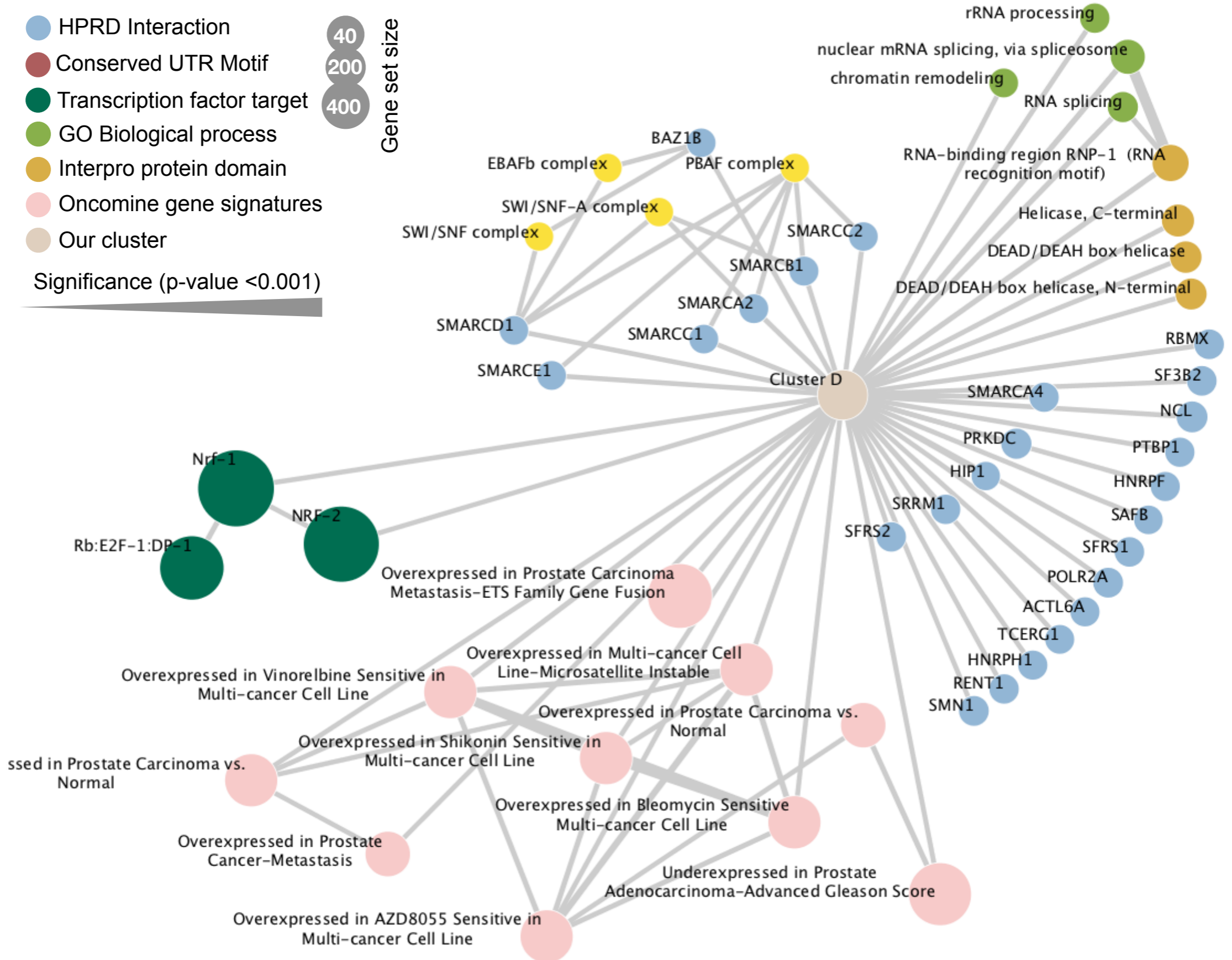

E

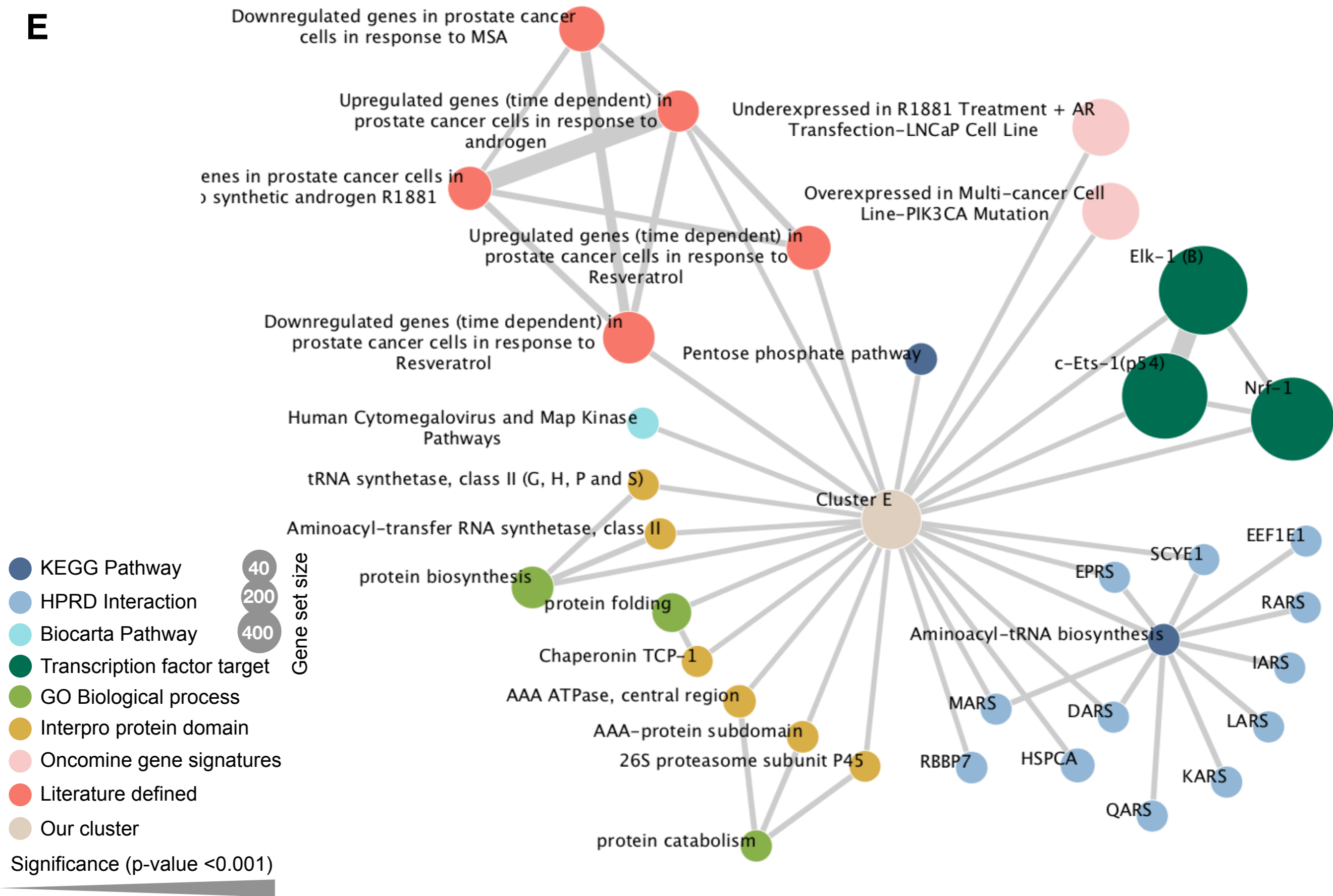

Supplement: Supplementary file 1 [file biomedicines-09-01877-s001.zip › Figure S5.pdf]

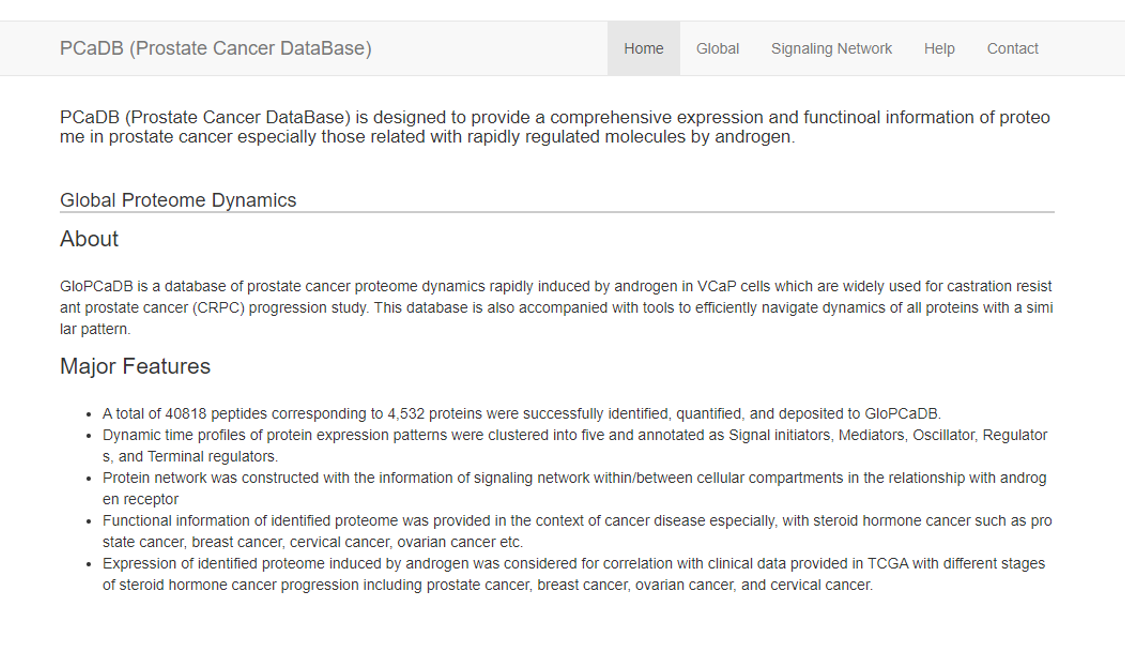

Supplement: Supplementary file 1 [file biomedicines-09-01877-s001.zip › Figure S6.tif]
